# A Decision Support System for preclinical assessment of nanomaterials in medical products: the REFINE DSS

**DOI:** 10.1007/s13346-022-01145-2

**Published:** 2022-05-10

**Authors:** Alex Zabeo, Fabio Rosada, Lisa Pizzol, Fanny Caputo, Sven Even Borgos, Jeremie Parot, Robert E. Geertsma, Joost Jacob Pouw, Rob J. Vandebriel, Oihane Ibarrola Moreno, Danail Hristozov

**Affiliations:** 1 GreenDecision Srl., Venezia, Italy; 2 Department of Biotechnology and Nanomedicine, SINTEF Industry, Trondheim, Norway; 3 Centre for Health Protection, National Institute for Public Health and the Environment (RIVM), Bilthoven, The Netherlands; 4 BioKeralty Research Institute AIE, Parque Tecnológico de Álava, 01510 Miñano, Spain

**Keywords:** Nanomaterials, Decision Support System, Intelligent testing strategy, Safety assessment

## Abstract

The application of nanomaterials in medicine has led to novel pharmaceuticals and medical devices that have demonstrated a strong potential for increasing the efficacy/performance and safety of therapeutic and diagnostic procedures to address a wide range of diseases. However, the successful translation of these technologies from their inception (proof-of-concept) to clinical practice has been challenged by substantial gaps in the scientific and technical capacity of R&D companies, especially SMEs, to keep up with the ever-evolving regulatory expectations in the emerging area of nanomedicine. To address these challenges, the EU Horizon 2020 project REFINE has developed a Decision Support System (DSS) to support developers of nanotechnology-enabled health products in bringing their products to the clinic. The REFINE DSS has been developed to support experts, innovators, and regulators in the implementation of intelligent testing strategies (ITS) for efficient preclinical assessment of nanotechnology-enabled health products. The DSS applies logical rules provided by REFINE experts which generate prioritized lists of assays to be performed (i.e. ITSs) for physicochemical characterisation and for immunotoxicological endpoints. The DSS has been tested against several case studies and was validated by internal project experts as well as external ones.

**Graphical abstract:**

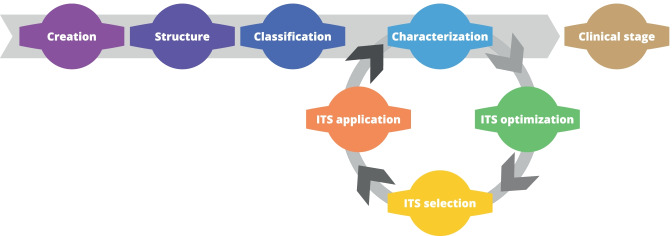

**Supplementary Information:**

The online version contains supplementary material available at 10.1007/s13346-022-01145-2.

## Introduction

Formulation of medicinal products using biocompatible NMs, the so-called nanomedicines, has the potential to improve a drug’s therapeutic index in various ways, e.g. improved pharmacokinetics and biodistribution. The key advantage of the first clinically successful nanomedicine, Doxil^®^ (liposomal doxorubicin), over the non-encapsulated parent drug has been the improved safety due to lower off-target cardiotoxicity, leading to an enlarged therapeutic window. Recently, NM formulation in lipid nanoparticles (LNPs) was the key enabling technology that made the mRNA-based Covid-19 vaccines possible. In medical devices, the application of NMs can impart properties that give significant therapeutic or diagnostic benefits, as in the case of Hensify^®^ (nanoparticulate hafnium oxide) used for radioenhancement in solid tumors [1].

Following the introduction into the clinic of Doxil^®^ in 1995, there was an expectation of a wave of NHPs. This has materialized more slowly than expected, and capacities for preclinical testing have been seen as a bottleneck. Evaluation of quality, safety, and efficacy of NHPs for medical applications follows the same fundamental principles as the evaluation of medical products without NMs. Nevertheless, the implementation of experimental assays frequently requires both significant adaptation of existing methods and the use of completely novel assays. Two examples are the measurement of particle size and size distribution, and the release kinetics of the active pharmaceutical ingredient (API) from the nanoparticle carrier. As many of these assays are highly specialized and technically demanding, a need was seen to provide infrastructures to support NHP developers in their acquisition of the preclinical safety data needed to go towards clinical testing of their new NHPs. This led to the establishment of the Nanomedicine Characterization Lab (NCL) in the US in 2004, and subsequently to the European Nanomedicine Characterisation Laboratory (EUNCL) as a H2020 project in Europe in 2015. Characterization capacities could be roughly classified into three principal categories; physicochemical, in vitro toxicity (cytotoxicity, hematotoxicity, immunotoxicity), and in vivo toxicity (animal experiments). To meet the complexity of the task at hand and the extreme variety in NMs investigated, and to perform rational and appropriate characterization, it became clear that in-depth scientific knowledge was needed to choose the right assays—in the right sequence—and tailor their application to each NM [2]. This applies even down to the single measurement endpoint, e.g. particle size, as recently published by scientists from NCL and EUNCL [3].

Despite recent advances in both knowledge and technology, clear gaps exist in the portfolio of available methodologies for quality and safety assessment of NMs used in health products. Scientists from the REFINE project recently published a review of needs and priorities for method development and standardization, related to regulatory information needs [4]. The identified methodological gaps and needs could be grouped into three categories, which were (1) nanomaterial-specific method adaptation, (2) validation and standardization of methods in early stages of development, and finally (3) development of additional methods in those areas where no or very few methods are currently available.

All of the above points to a need to develop and implement robust, transparent, science-based principles on how to select characterization methods towards the currently optimal testing strategy for any given NM. This would support NM developers and other stakeholders in advancing more candidate NHPs towards the clinic. In the REFINE project, we have been developing such principles in the form of intelligent testing strategies (ITS), implemented in the form of a Decision Support System (DSS).

A Decision Support System (DSS) is a computer-based software tool used to support complex decision-making and problem solving [5]. Specifically, when public authorities or companies must manage complex problems, a DSS facilitates their decisions by providing a framework that efficiently delivers ideas, best practices and searchable resources. DSSs can offer data collection functionalities, logical and quantitative analyses and can facilitate the communication of results by means of easy-to-understand charts, graphs and figures. DSSs also allow the integration of different types of information. They can include integrative methodologies, such as risk-cost–benefit or technological assessments and can evaluate and rank management alternatives by implementing decisional methodologies such as, for example, Multicriteria Decision Analysis (MCDA). In the nano safety area, different DSSs have been developed for the occupational risk assessment of NMs; these are, for example, the BIORIMA DSS designed to estimate occupational and environmental risks of NMs used in Medical Devices and Advanced Therapy Medicinal Products along their life cycle [6] and the SUNDS system for occupational risk assessment of NMs used in the industrial sector [7–9]. Also in the medical sector, ICT tools, including modelling and simulation tools, have grown to become a reliable approach to better understand and optimize key decisions related to safety, efficacy, dosing, and special target populations [10]. However, few DSS tools have been developed to support producers of medical devices and medicinal products to undertake the process for the market authorisation of their products. Most of them are clinical decision support (CDS) systems related to medication prescribing, design of alerts, reminders, and other types of intervention [11, 12]. For medicinal products, DSSs are developed for pharmaceutical formulation optimization [13], to estimate the probability of adverse drug reactions [14], or to support formulation scientists modelling approaches for different biological scales [15, 16]. In pharmacovigilance, additional tools have been developed to automate routine work and to balance resource use across safety risk management and other pharmacovigilance activities. Three categories of intelligent automation systems, ranging from rule-based systems to dynamic AI-based systems have been identified and listed by Huysentruyt et al. [17]. These categories are “rule-based static systems,” “AI-based static systems,” and “AI-based dynamic systems.” Additionally, Rathore et al. [18] provided a review of the interface between Knowledge Management (KM) and Quality by Design (QbD)-driven biopharmaceutical production systems as perceived by academic as well as industrial viewpoints. It includes a comprehensive set of 356 publications addressing the applications of KM tools to QbD-related tasks, including a specific class related to intelligent process management in continuous pharmaceutical operations and intelligent decision support in pharmaceutical development. However, none of the reviewed ICT tools has been developed to support the pre-clinical safety testing for the market authorisation of NHPs, whether medicinal products or medical devices. Accordingly, one of the objectives of the REFINE project is to develop a DSS which supports developers of nanotechnology-enabled health products in bringing their products to the clinic. This tool is complementary to those already developed and can be used synergically to support producers of medical devices and medicinal products to undertake the process for the market authorisation of their products.

The objective of this paper is to present the methodology behind the development of the intelligent testing strategies (ITS) for physicochemical characterisation and immunotoxicological pre-clinical safety assessment of NMs used in medicinal products and medical devices and its implementation within the REFINE DSS.

## Methodology

### Overview of the methodology

The Refine DSS is a tool that supports the user to set up their pre-clinical safety testing for the market approval of NHPs, whether medicinal products or medical devices.

The DSS guides the user through several steps in order to gain more knowledge about the NHP under development. The process is based upon the ITS loop, i.e. a series of iterative assessment steps that the user takes several times which allows to acquire more knowledge about the NHP in the most efficient way.

The aim of the DSS is to guide the preclinical assessment of a NHP and by that get the largest amount of useful and necessary information in the most efficient way to finally reach the clinical stage. To do so it is necessary to apply a series of assays, belonging to different modules. This process can be optimized by performing a selection of modules and category endpoints to be measured based on the properties of the NHP and of the embedded NM, and a selection and prioritization of the assays for each selected category endpoint based on assays’ features.

The first NHP’s property that the model takes into consideration is whether the NHP is a medical device or a medicinal product. This information is relevant since modules to be assessed have specific features for one or the other category. Other specific properties which are considered in the selection of modules are related to how the NHP is used, with properties like contact type or contact duration for the MD and clinical indication and administration route for the MP. Such selection of modules based on NHP’s properties was derived from ISO as well as ICH documents. For the physical and chemical characterisation modules, specific rules were developed during multiple sessions with the available experts working in the REFINE project. For the immunotoxicity module, specific rules were derived from previous work of partners in the project, as reported by Giannakou et al. [19].

As far as it concerns the embedded NM, several category endpoints are defined and considered for each module. These category endpoints define intrinsic properties of the NM, and they can be either qualitative or quantitative. For example, in the physical characterisation module, some of the quantitative properties considered are particle size diameter, particle density, polydispersity, and surface charge; some of the considered qualitative properties are formulation (liquid or powder), particle classification and some immunotoxicity module’s endpoints such as sts—haematological changes, sts—alterations in immune system, sts—changes in serum globulins and others.

The qualitative properties are aspects of the NM that are non-measurable; they are usually known a priori. These properties constitute the initial batch of information provided to the DSS about the NM. The quantitative properties are instead measured through specific assays. The application of these assays produces output values that belong to the continuous space. Nevertheless, a minor selection of aspects is only qualitative as related to the interpretation of quantitative results coming from complex assays, such as determining if T-cell-dependent antibody response is present or not in the immunotoxicity module.

As mentioned above, each assay produces numerical values; these values are associated with endpoints. This type of information is difficult to model and formalize in the knowledge base. In fact, the goal is to have values representative of each category endpoint, but there are no established ways to convert endpoint values in the same category to a single representative result.

A set of qualitative classes was then assigned to each category endpoint. This class division helps in two ways:

- Logical rules are also defined in terms of classes. In this way, the provided data and the rule system rely on the same structure.

It is easier for the user to input the results into the system, as it is just a matter of identifying in which range the result falls.

This division was made in a meaningful way, taking into consideration the operative range of the most common assays. For example, for *surface charge, the following* classes have been defined: positive (zeta > 10 mV), neutral (−10 mV < zeta < 10 mV), and negative (zeta <  −10 mV).

As stated before, rules for assays’ selection are based on classes; such rules are defined through the logical AND, OR, and NOT operators. These simple operators can be combined to form complex sets of rules that apply to each endpoint. For example, applicability rules in the physicochemical module for the nanoparticle tracking analysis (NTA) assay are:

$$\left[\begin{array}{c}\mathrm{Refractive\;index }=\mathrm{ low\;RI }\left(\mathrm{RI }< 1.6\right)\\ \mathrm{AND}\\ \mathrm{Particle\;size\;diameter }\left(\mathrm{lower\;bound}\right)= 60 \\ \mathrm{AND}\\ \mathrm{Particle\;size\;diameter }\left(\mathrm{upper\;bound}\right)= 1000\end{array}\right]\mathrm{OR}\left[\begin{array}{c}\mathrm{Refractive\;index }=\mathrm{ high\;RI }\left(\mathrm{RI }\ge\;1.6\right)\\ \mathrm{AND}\\ \mathrm{Particle\;size\;diameter }\left(\mathrm{lower bound}\right)= 30 \\ \mathrm{AND}\\ \mathrm{Particle\;size\;diameter }\left(\mathrm{upper\;bound}\right)= 1000\end{array}\right]$$

while as an example of the immunotoxicity module, for the OECD 442C assay related to in chemico skin sensitisation rules are:

$$\left[\begin{array}{c}\mathrm{Route\;of\;administration}=\mathrm{Dermal}\\ \mathrm{AND}\\ \mathrm{Dermal\;Systemic\;exposure}=\mathrm{NO}\end{array}\right]$$

After having established the structure of the knowledge base and the selection of assays to be measured, a way of prioritizing assays is necessary to create an efficient testing strategy. A set of assays’ characteristics was selected to drive this prioritization: duration, cost, resolution, and expertise. Their meaning is respectively:

- Duration indicates the time spent between when the assay starts and when its results are ready. Short duration is preferred, to speed up the development process.
- Cost expresses the overall cost required to run the assay. This includes for example equipment required, consumable materials, and personnel involved.
- Resolution indicates the smallest change that can be measured (or detected); higher resolution is usually preferable.
- Expertise represents the level of experience required by the personnel to run the assay, the lower the better since required high expertise might impact costs, but also duration since a low availability of experts might delay the execution.

Each characteristic has been divided into three generic classes based on relative impacts among other assays measuring the same category endpoint as established by involved REFINE experts. To each class, a numeric score was associated where 1 stands for “low” and 3 for “high.”

The prioritization of valid assays is obtained by integrating the above-mentioned characteristics’ scores. Values for duration, cost, and expertise are considered better when low, while resolution is considered better when high. The formula to obtain the priority score is:

$$\mathrm{priority}=\mathrm{duration}+\mathrm{cost}+\left(3-\mathrm{resolution}\right)+\mathrm{expertise}$$

The model then takes advantage of selection and prioritization of the assays to guide an iterative investigation on the NHP. It starts with an initial basic amount of, mostly qualitative, information and, based on that, as explained in the previous points, it performs a first selection and prioritization of meaningful assays. The user can then select an assay, perform it, and include its results in the system. The new information will be used by the model, which can now perform a more precise selection and prioritization. This series of iterations results in an ITS (intelligent testing strategy), and it is repeated until there is enough information to move on to the clinical stage.

### Implementation of the methodology

**Fig. 1:**
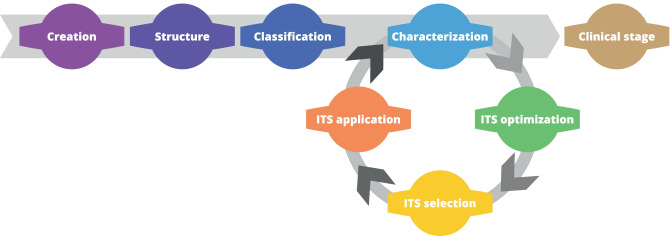
Stages of DSS

We have implemented the methodology in a web application that guides the user through the collection of information about the NHP. The web application allows to create and share NHPs with other users. This creates a collaborative environment and enables inputs from multiple sources. This way the development of the NHP can progress faster through its various constituting stages as shown in Fig. 1 and reported in the sections below.

#### Creation

The first phase is the creation of the NHP. During this phase, the user is asked to input basic information about the NHP such as name, description, and some basic technical information. The basic information starts with type which identifies whether the NHP is a medical device or a medicinal product. Afterwards, more information is asked based on the type, medical devices are characterized by device category, contact type, and contact duration; medicinal products by clinical indication and administration route.

#### Structure

In this phase, the user can define the structure of the NM embedded in the NHP by specifying its components and layers. Specifically, for MP it is possible to define components by their category (APIs, excipients, buffers, and impurities) while for the MD, generic components are defined. Each component can be appointed to one or more layers (e.g., core, shell, coating), and their concentration and CAS number can be specified. A specific ITS is then provided for each component separately as well as for the entire NM.

#### Classification

**Fig. 2:**
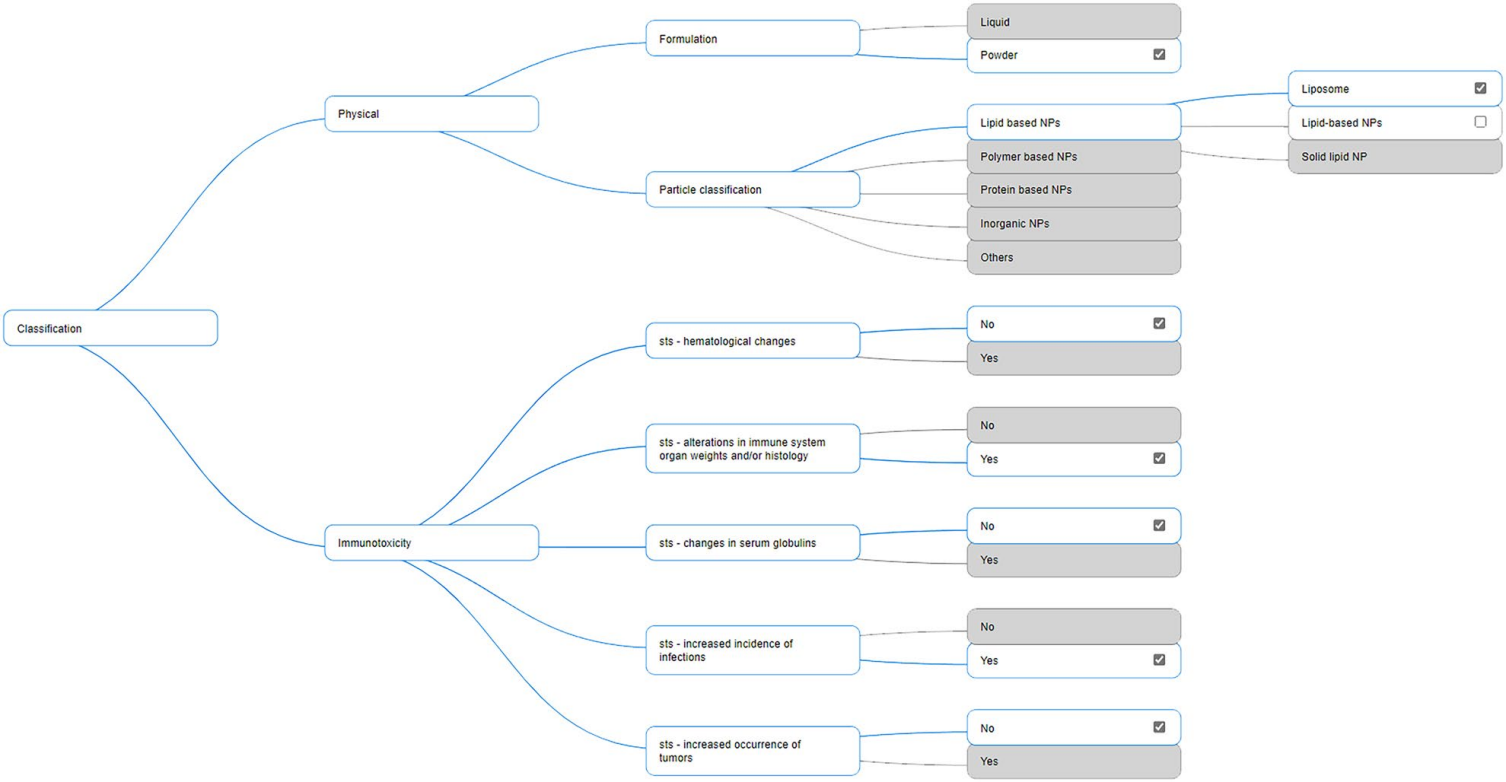
Classification tree

In the classification phase, the user is asked for qualitative known information about the NHP and the embedded NM which is subsequently used by the ITS. The type of information asked during this stage does not require any measurement; it is basic information about physicochemical and immunotoxicology properties the user should already be aware of. For example, the user is asked if the formulation is either liquid or powder, or if the sts—haematological changes are present or not. The system asks for this information by presenting a tree of properties (see Fig. 2). This tree has embedded rules that guarantee the selected choices are consistent (e.g., it is not possible to select both liquid and powder in the formulation branch).

#### Characterization

**Fig. 3:**
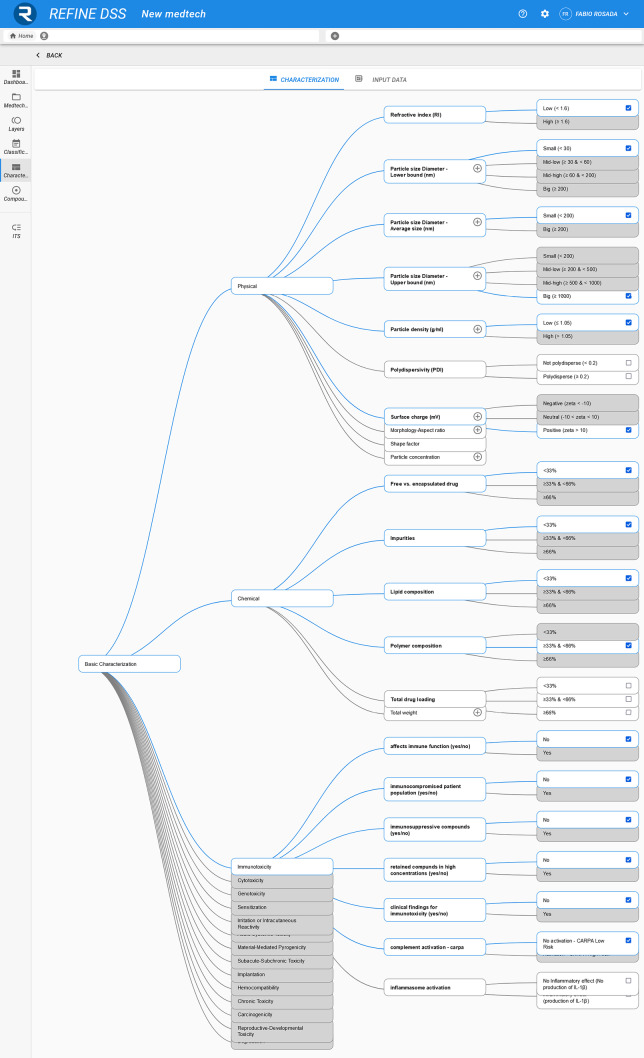
characterization tree

In the characterization phase, NM quantitative information is required, which comes from the application of specific assays. This stage is divided into two sections: characterization and input data. Characterization is presented as an information tree (see Fig. 3), where, for each property, it is possible to select a specific class of results (i.e., a range of values). For example, in the physicochemical module, particle size diameter—lower bound is divided in 4 classes: small (< 30 nm), mid-low (> = 30 and < 60 nm), mid-high (> = 60 and < 200 nm), and big (> = 200 nm), while in the immunotoxicity module, affects immune function comprises the classes yes or no. Input data consists of a table where it is possible to add assay results with the related conditions, media, and experiment repetitions. This table will serve as a guide to fill in the characterization’s information tree which is the one guiding the ITS. With future updates of the DSS, the input data values might be used to automatically select proper characterization classes.

#### ITS optimization, selection, and application

The last phase consists of the application of the ITS; it is the core of the DSS and should be repeated several times until enough information is gained to proceed to the clinical assessment phase. The ITS scans the available assays and, based on specific rules, it filters out the assays which are not suitable for the NM (e.g., if the particle size is bigger than 1000 nm, then the DLS assay cannot be applied). It is important to note that the DSS allows users to create new assays by specifying their characteristics, these are automatically involved in the ITS. The ITS will then present, for each module and endpoint, a prioritized list of possible assays that can be used to further improve the knowledge of the NHP. Assays are presented separately according to their resolution (i.e. low resolution assays are separated from medium–high resolution assays) and prioritized by their characteristics such as duration, cost, resolution, and expertise. It is now possible to select the best assay and apply it in real life. Once new assays’ results are added to characterization, the ITS takes into account the new information to further optimize the recommendation possibly proposing different assays.

### Software development

The DSS was developed as a reactive web application by applying a variation of the widely used MEAN (MongoDB, Express.js, Angular, and Node.js) stack software bundle application. Variations of the standard MEAN stack relate to the use of the Meteor JavaScript framework instead of Express.js and React instead of Angular as the selected user interface management library. To supply dynamic visualisations and charts the D3.js library was utilized.

## Results and validation

The presented methodology was implemented into a Decision Support System as a reactive web application which is publicly available at https://refinedss.eu. To test and validate the developed physicochemical and immunotoxicology modules of the REFINE DSS, the DSS has been tested on four case studies and validated through the presentation of the results and applied rules to panels of internal and external experts in dedicated workshops.

### Case studies application

One medical device (dextran-coated iron-oxide nanoparticles) and three medicinal products (liposomes, iron carbohydrates, and PACA) were utilized as case studies for testing the DSS application as described in the following sections. They cover both the inorganic and organic categories. Moreover, two case studies have been already approved, while the remaining case studies are still in the approval process. The heterogeneous characteristics of the case studies allowed to test the system in multiple settings.

#### Dextran-coated iron-oxide nanoparticles (IONP)

BK-MNP is a medical device; it is an aqueous suspension of multicore nanoassemblies comprising magnetite Fe3O4 (CAS: 1317–61-9) and/or maghemite γ-Fe2O3 (CAS: 1309–37-1) cores in a dextran matrix, the dextran having a molecular weight of 40 kDa and chemical formula H(C6H10O5)xOH (CAS: 9004–54-0). Multicore nanoassemblies are characterized by nanoparticles in exchange coupling, causing a collective magnetic order [20].

In general, magnetite and maghemite particles are the most commonly used magnetic materials in medicine for diagnosis and therapy and are generally well tolerated. Their magnetic properties allow their application for hyperthermia therapy taking advantage of their remotely controlled accumulation by means of an external magnetic field. Maghemite: γ-Fe2O3 can be considered fully oxidized magnetite. Similar to magnetite, maghemite exhibits ferrimagnetism at room temperature (saturation magnetization up to 80 emu g^−1^) and presents a cubic structure.

BK-MNP has been designed to meet the requirements of the Medical Device Directive 93/42/EEC and amendments up to and including 2007/47/EC as a Class III medical device and is manufactured under ISO 13485:2016 controls. Before being removed from the ISO Class 8 clean-room, BK-MNP is filled into sterile size 2R glass vials in 0.5 ml aliquots, and sealed with rubber septa and crimp tops. Post-production gamma sterilisation of BK-MNP is performed with up to 25 vials packaged per 49-well Cryobox container, and with no two vials placed in adjacent wells (so as to avoid overshadowing effects).

#### Liposomes (LP)

Doxil^®^, the first FDA-approved nano-enabled medicinal product (1995), is a PEGylated liposome encapsulating doxorubicin into a lipid bilayer in a “liquid ordered” phase composed of phosphatidylcholine, and cholesterol. It is administered intravenously and is currently used to treat AIDS-related Kaposi’s sarcoma, breast cancer, ovarian cancer, multiple myeloma, and other solid tumors [21].

Doxil’s successful development opened the way to a major improvement in tumour therapy and it served as a gold standard in nanomedicine. It also showed for the first time the importance of understanding the physicochemical properties of a nanocarrier and that controlling its impact on safety and efficacy is crucial to the successful development of such a complex drug product. Doxil was chosen as the example of a well-known and well-characterized liposomal product formulation that served as the first proof of concept of the DSS system and as an example for evaluating the testing strategy of other liposomal formulations.

#### Iron carbohydrates (IC)

Feraheme^®^ [22] (generic name ferumoxytol) is a medicinal product. It received marketing approval from the FDA in June 2009 as an iron replacement product indicated for the treatment of iron deficiency anemia in adult patients with chronic kidney disease. It is administered intravenously. The API ferumoxytol consists of an iron oxide core (maghemite, γ-Fe2O3) surrounded by a carbohydrate coating [23, 24]. Ferumoxytol was chosen as one of the case studies because it has been on the market for over a decade, and therefore, information on the physical and chemical properties of the product is widely available and easily accessible.

#### Poly (alkylcyanoacrylate)

Poly(alkylcyanoacrylate) are biodegradable polymers built around a cyanoacrylate polymer backbone, where the choice of the alkyl side groups impacts important aspects like drug encapsulation, degradability, and cytotoxicity. The polymer used in REFINE was poly(ethylbutyl cyanoacrylate) (PEBCA). The PEBCA nanoparticles were synthesized under aseptic conditions at SINTEF by miniemulsion polymerization; emulsion was produced by sonication. Encapsulation of the antineoplastic drug cabazitaxel makes the Poly(alkylcyanoacrylate) (PACA) a medicinal product that is injected intravenously. The dispersions were dialyzed to remove unreacted PEG. The size (*z* average), polydispersity index (PDI), and the zeta potential of the NPs in phosphate buffer are measured by dynamic light scattering (DLS) and laser Doppler Micro-electrophoresis. To calculate the amount of encapsulated drug, the drug was extracted from the particles and quantified by liquid chromatography coupled to mass spectrometry (LC–MS/MS).

#### Case studies results

In all the applied case studies, the proposed ITS was considered to be in line with the expectations of the involved experts confirming that the implemented decisional logic follows what is usually applied by practitioners. More specifically, the most discriminatory properties for excluding unsuitable assays were formulation, particle classification and size (physical), and compound class and molecular weight (chemical) for *IONP*; particle classification (physical) and molecular weight (chemical) for *LP*; particle size (physical) and molecular weight (chemical) for *IC* and *PACA*. As far as the immunotoxicity module, the selected classification and characterization classes did not trigger specific rules and therefore the initial list of immunotoxicity assays was proposed. This is related to the smaller number of existing rules in the immunotoxicity module compared to the physicochemical one.

**Fig. 4:**
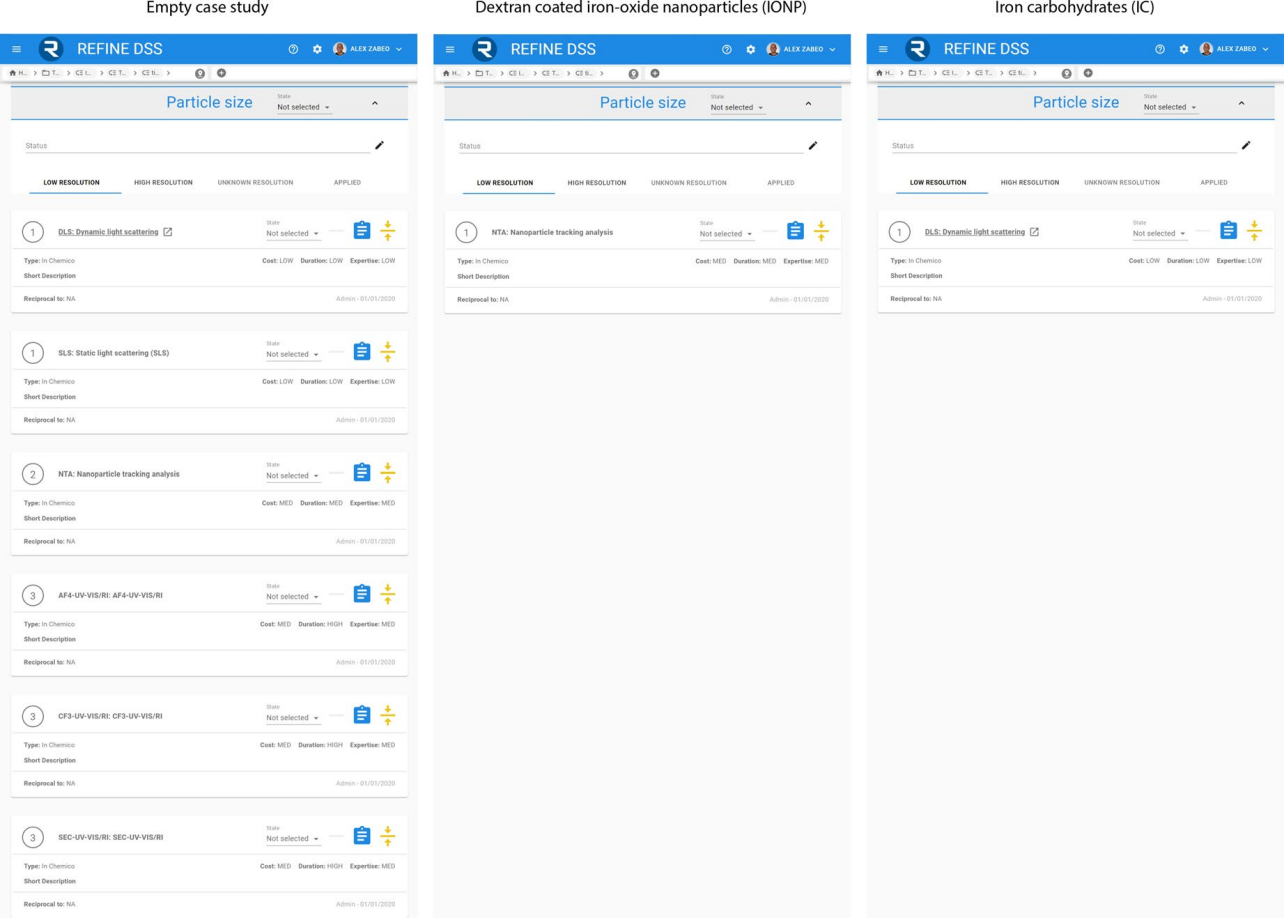
Comparison of DSS results between an empty case study, IONP and IC. The absence of information in the empty case study results in the complete list of available assays while for IONP and IC most of the assays are inapplicable due to their characterization values

A comprehensive description of case studies’ results is outside the scope of this manuscript. In Fig. 4, an example of the REFINE DSS results is provided, showing a comparison between an empty case study, *IONP*, and *IC* for the particle size endpoint, low resolution assays. In the empty case study, where no prior information is supplied to the system, all available assays are proposed to the user as none of them is known to be unapplicable. In this case the prioritization proposes to start with *DLS* or *SLS* which are the most comprehensive and easily applicable assays. When some characterization data are present, like in the other two case studies, unapplicable assays are filtered out from the ITS list leaving only *NTA* for *IONP* and *DLS* for *IC*. *AF4-UV–VIS/RI, CF3-UV–VIS/RI*, and *SEC-UV–VIS/RI* were discarded for both *IONP* and *IC* because of their particle classification as a Metal oxide nanoparticle and *SLS* because of a small particle size diameter. In *IONP*, *DLS* was discarded because of polydispersity while in *IC NTA* was discarded because of particle size diameter being very small.

### Validation with experts’ panels

The results of the case studies applications have been presented and discussed in internal meetings with REFINE experts in physicochemical characterisation as well as with external experts who participated in dedicated workshops. The aim of the workshops was to collect experts’ feedbacks on the suitability, effectiveness, robustness and usability of the physicochemical modules of the REFINE DSS which have been presented following the liposomes case study. The immunotoxicity module was not considered in this round of validations as not completely developed by the time validation took place; it will be the focus of a future validation which is behind the aims of this paper.

#### First workshop, internal experts

Nine experts in physicochemical characterisation attended the first internal workshop event. Along with questions and comments collected during the meeting, specific questions were asked using the SLIDO tool (www.sli.do). The list of questions is reported in Supplementary material PART 1 along with the provided answers. As a first reaction, all responding experts felt that the methodology developed for the prioritisation of physicochemical tests is suitable for its purpose. However, 4 experts found that additional aspects needed to be considered in the prioritization methodology. This included numerous practical aspects of the assays that are relevant as well as specific measurement purposes that can be different at different stages of development. There was no opposition to the aspects currently considered in the methodology. Regarding usability, it was not easy for everyone to follow the logical structure of the methodology. This was because there was not enough detail of what could be handled in the system and how it worked. Moreover, the participants found that the tool was too complex and overly expert-oriented. Therefore, it was not easy to be used by non-experts or by experts on only part of all aspects considered in the DSS. Specifically, it was suggested to make clearly available the list of available methods included and assessed by the DSS along with the endpoints that they can measure, and the list of decisions made by the tool to filter/select the different proposed methods.

#### Second workshop, external experts

The suggestions collected during the first workshop were implemented in the REFINE DSS before the second workshop was held, where five external experts in physicochemical characterisation actively participated in the assessment and evaluation of the developed REFINE DSS. Along with questions and comments collected during the meeting, specific questions were asked using the SLIDO tool. The list of questions is reported in supplementary material PART 2 along with the provided answers.

All participants agreed that the modules of the REFINE DSS for the prioritisation of physicochemical tests are suitable for the purpose. Three experts identified additional aspects to be considered. These aspects were the consideration of different stages of product development (R&D, production, quality control for batch release), different measurement purposes (investigate product stability, determine batch to batch variability, etc.) and different scenarios (product containing biologics, product containing a novel excipient, etc.). This means defining measurement priorities also according to specific requirements associated with a different scenario. Indeed, these aspects could affect the quality control procedures and the need for standard methods (as in the case of regulatory approval) or the possibility to use innovative methods that may not be yet standardized, as in the case of the research and development scenario. Accordingly, the assays included in the DSS should be classified based on their maturity, as defined in the recent REFINE publication from Halamoda-Kenzaoui et al. [4]. Besides these aspects, there was no opposition to the aspects currently considered in the methodology. Regarding usability and how clear is the DSS structure, most of respondents found it easy to understand how the DSS works. Additionally, the DSS functionality which allow users to include the specific instruments and methods available in their labs or provided by their service providers was recognized as particularly useful.

Finally, one additional aspect that should be included in the DSS is the reference to the SOPs, or standard test methods that could be associated with each assay, and is applicable to a specific particle class with specific conditions (e.g. sample preparation). This is still missing in the current version of the DSS, but it is an important aspect that should be added in the next version. Additionally, selection criteria will be added to describe the maturity of the specific assay to be used, e.g. whether it is validated and accepted by regulators.

#### General outcomes from the two workshops

Both the internal and external experts agreed that the DSS users can be SMEs developing NMs, large pharma and MD companies developing NMs, regulatory agencies, and the scientific community. However, it seems that SMEs could be the most likely users.

Similarly, the two workshops identified that the main obstacle for the use of the REFINE DSS is the collection and generation or even lack of data. Specifically, one expert explained that an important point is the involvement/inclusion of stakeholders and especially of regulators. Indeed, a central point will be to understand to what extent the developed DSS could be accepted by regulators.

One additional meeting with external experts in physical–chemical characterisation of iron carbohydrates has been organised to specifically validate the physical–chemical intelligent testing strategy for iron carbohydrates.

The experts participating in the two workshops and the external meeting stated that the testing strategies proposed by the REFINE DSS were in line with the expectations and they did not identify any specific issues.

## Discussion and conclusions

In this paper, a methodology for a robust, transparent, science-based, and regulatory-based selection of characterization methods for physicochemical and immunotoxicological pre-clinical safety assessment of NMs used in medicinal products and medical devices has been presented along with its implementation within a web-based DSS.

The developed methodology consists of logical rules based on classification and characterization values which are iteratively applied as new information arises. The rules are used to select which assays are applicable given the current conditions. The remaining assays are then prioritized according to a scoring system based upon a selected set of assay’s characteristics; this prioritized list of assays constitutes the ITS. This methodology was implemented into a Decision Support System as a reactive web application which is publicly available at https://refinedss.eu.

The REFINE DSS was applied to four case studies and validated through the involvement of internal and external experts.

Application to case studies demonstrated that in all cases, the proposed ITS was in line with experts’ expectations. The properties with the highest influence on the ITS for the assessed products were mostly related to formulation, particle classification, and molecular weight.

The REFINE DSS was positively evaluated during validation steps where (i) all experts agreed that the modules of the REFINE DSS for the prioritisation of physicochemical tests are suitable for their purpose, (ii) there was no strong opposition to any aspects present in the DSS, (iii) it was easy for them to understand how the DSS works as well as to use the access pages and the different functionalities. Moreover, the results of the intelligent testing strategies were considered clear and easy to understand. This positive response was especially true for the second (external) webinar, while during the first (internal) webinar, some difficulties occurred in understanding the methodology behind the REFINE DSS. This was solved in the second webinar by (i) presenting the tool focusing on the liposomes case study, (ii) better contextualising the objectives of the developed prioritization methodology and related DSS, (iii) listing the results that this tool can provide and the benefits of using the DSS.

However, there are different aspects to be considered to improve the tool and to foster its sustainability. The first is a general technical aspect related to the usability of the DSS: it should be improved by providing a specific functionality that allows to effectively report how the prioritization of the available assays was performed, showing the list of “decisional rules” that the tool used to select and prioritize the proposed assays. Additionally, two minor technical improvements have been identified. First, it has been suggested to include information related to the maturity of the available assays. Indeed, this aspect could affect the quality control procedures and the need for standard methods (as in the case of regulatory approval) or the possibility to use innovative methods that may not be yet standardized, as in the case of the research and development scenario. The maturity classification could implement the classification recently defined by Halamoda-Kenzaoui and colleagues [4]. The second suggested technical improvement was to include different scenarios (e.g. product containing biologics, product containing a novel excipient) and measurement purposes (investigate product stability, determine batch to batch variability, etc.) as additional rules to guide the intelligent testing strategies.

Other improvement aspects are related to the future sustainability and use of the tool in real life. The first aspect to consider is the need that the tool is accepted by regulators so that it can be used in the compliance processes. Related to this objective, there is a challenge to ensure the sustainability and updating of the tool over time as science and regulatory expectations evolve.

In conclusion, we have developed a first version of a DSS that can support NHP developers and other stakeholders in advancing more candidate NHPs towards the clinic. Experience with this first version will allow the development of more sophisticated versions in the future. This concerns the integration of additional modules such as, for example, cytotoxicity, genotoxicity, hemocompatibility, carcinogenicity, and other relevant aspects to be consider in the pre-clinical safety assessment. As such, the current work contributes a theoretical framework that has value beyond the implementation of the DSS software, as it forms a science-based, transparent, and logically consistent approach to preclinical characterization of NHPs.

Indeed, the developed DSS is not able to cover all the requirements of the pre-clinical evaluation of medicinal products and medical devices. The provision of a full functional DSS for pre-clinical assessment of a nano-based medical technology was too ambitious to be reached in only one project. Accordingly, the REFINE DSS has been reassessed towards the delivery of a tool contributing to scientific knowledge in pre-clinical assessment, which can provide some functionalities which are fully implemented and properly tested by internal and external experts. Two modules reached these objectives: the chemical and physical modules. Additionally, the immunotoxicity module has been developed and applied, but it was not subject to a in dept testing by external experts as done for the chemical and physical modules.

## Supplementary Information

Below is the link to the electronic supplementary material.

Supplementary file1 (DOCX 20693 KB)

## Data Availability

The datasets generated during and/or analysed during the current study are available from the corresponding author on reasonable request.

## Glossary

Assays: are tests, whether in chemico, in vitro, in vivo, or in silico, which are applied to evaluate one or more endpoints.
Category endpoints: are generic measurable entities which can be measured through an Assay with a specific endpoint, e.g. the category endpoint particle size can be measured with the assay *DLS* which provides the specific endpoint hydrodynamic diameter (*Rh*). A category endpoint can generally be measured by various assays.
Endpoints: are specific measurable properties of the NM, e.g. hydrodynamic diameter (Rh).
Intelligent testing strategy (ITS): is the proposed sequence of assays that the DSS prioritizes for a specific NHP, depending on the NM in the NHP and assay characteristics.
Modules: represent macro categories of endpoints that can be measured in a NM or in one of its components, e.g. physical characterization, chemical characterization, cytotoxicity, etc. A module is composed of multiple category endpoints [25, 26].
Nanotechnology-enabled health product (NHP): is a final product which encompasses a nanomaterial (NM).
NHPs belong to one of the following nanotechnology-enabled health product types: Medical device (MD) and Medicinal product (MP).
