# Supplementary material for: A Decision Support System for preclinical assessment of nanomaterials in medical products: the REFINE DSS
Source: Drug Deliv Transl Res. 2022 May 10;12(9):2101–13. doi: 10.1007/s13346-022-01145-2 (PMC9089298; doi:10.1007/s13346-022-01145-2)
Supplement: Supplementary file 1 — Supplementary file1 (DOCX 20693 KB) [file 13346_2022_1145_MOESM1_ESM.docx]

Supplementary material

# Part 1: questions and answers for the First Internal Webinar

1. Do you think that the developed methodology for the prioritisation of physical-chemical tests is suitable for the purpose?


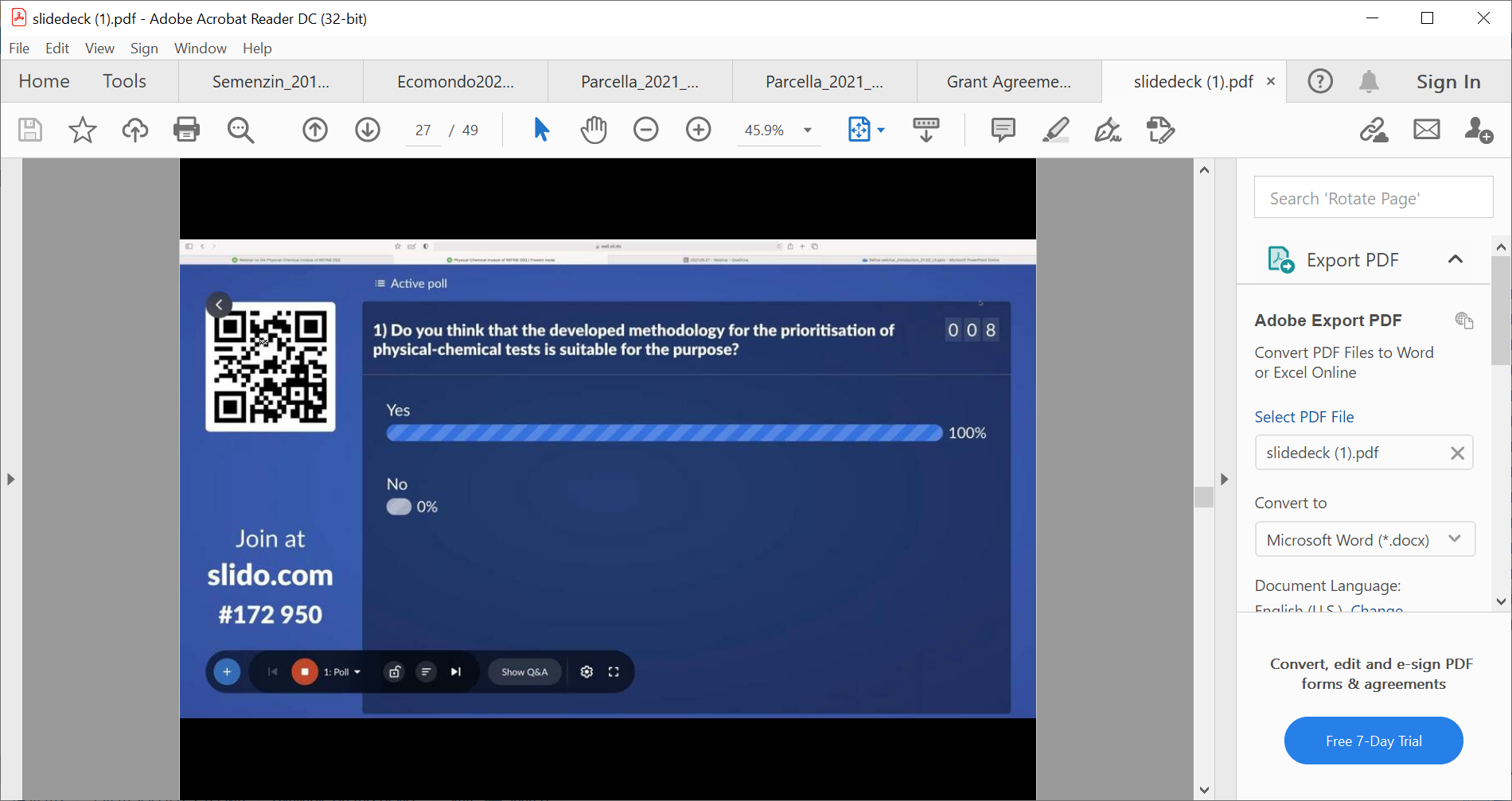


Figure 1. Answers to the first question

1. Can you identify additional aspects that need to be considered in the prioritisation methodology?


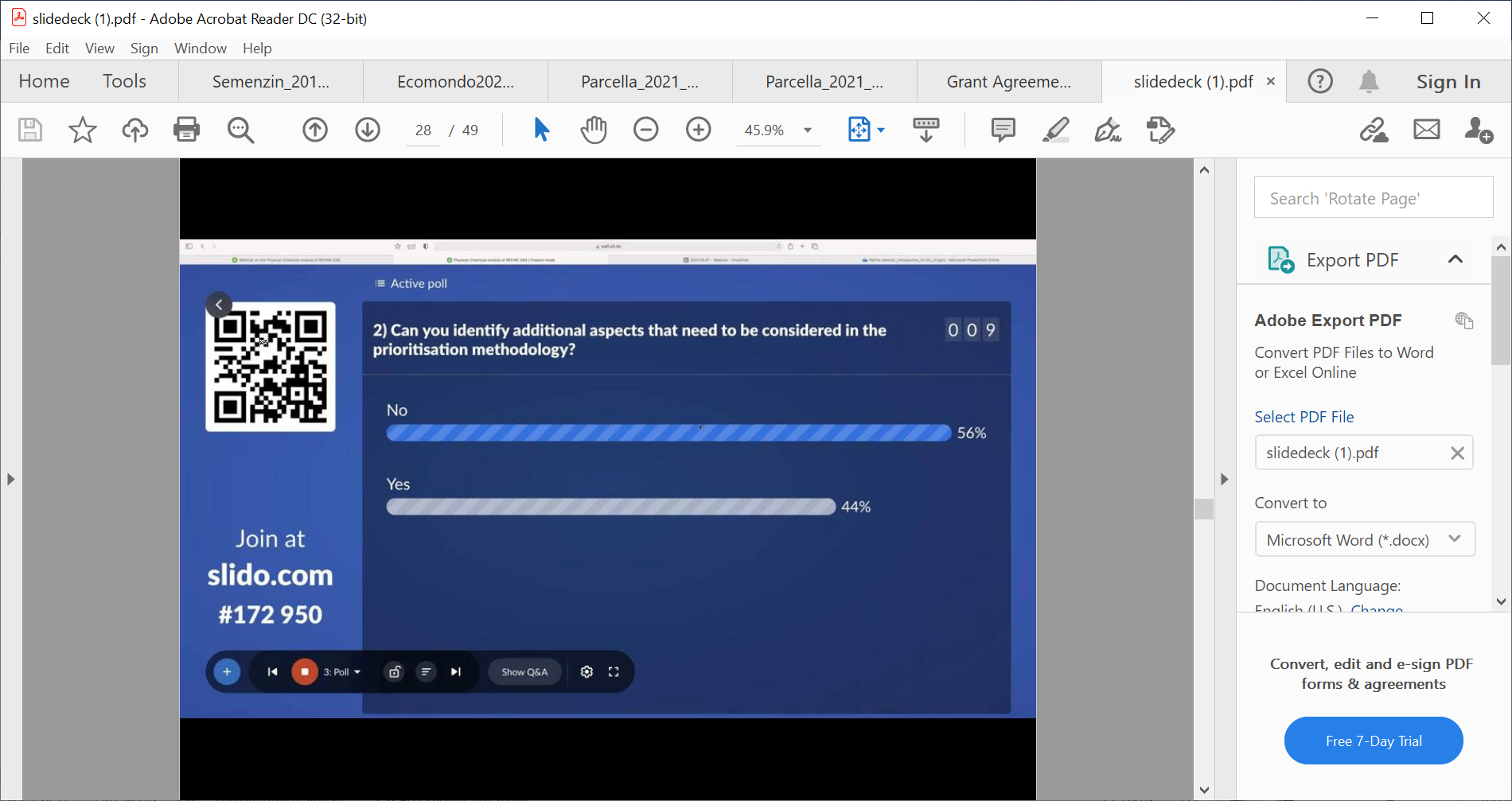


Figure 2. Answers to the second question


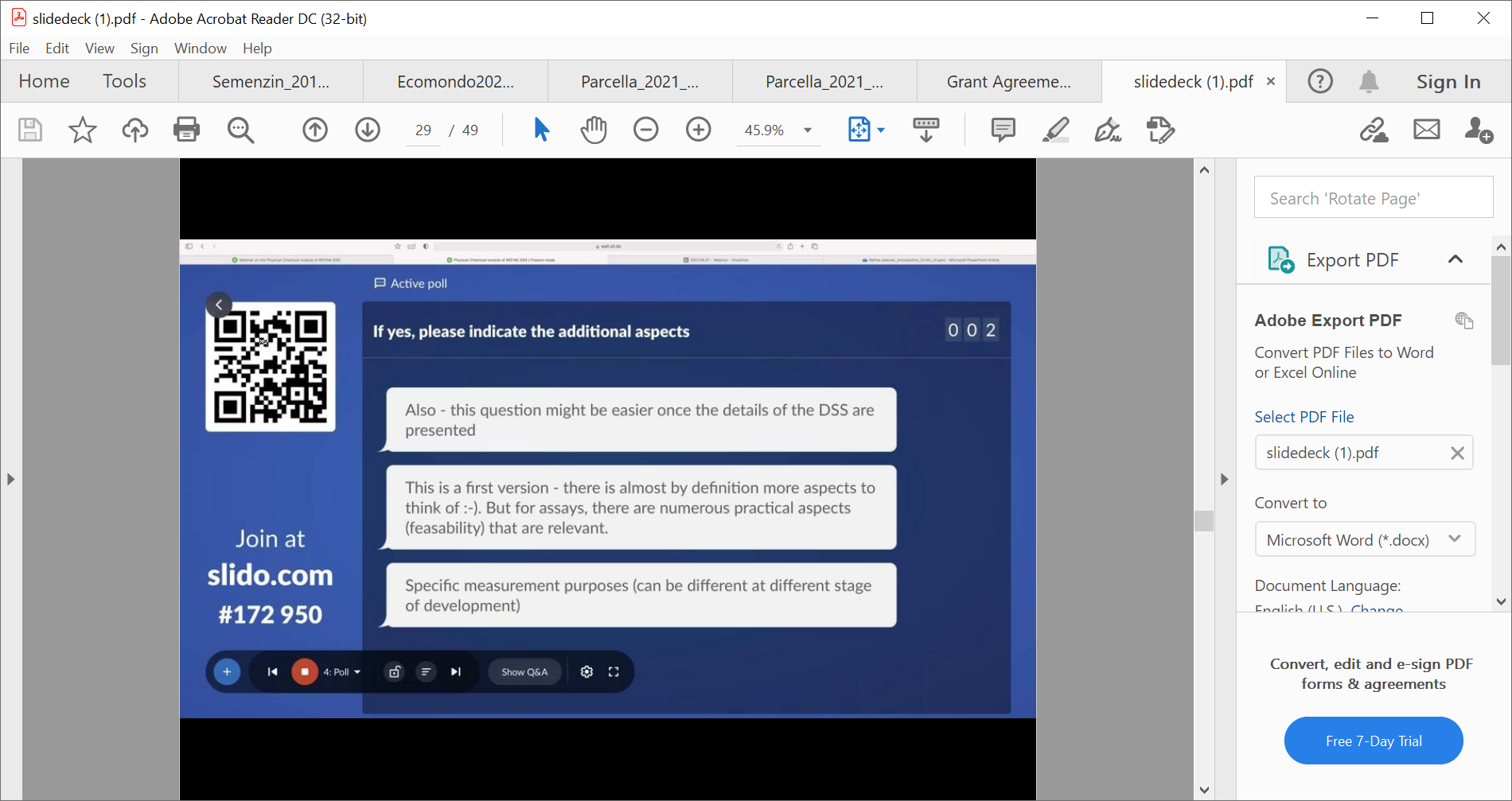


Figure 3. Additional aspects that should be included in the methodology.

1. Do you have strong opposition to specific aspects of the proposed methodology for the prioritisation of physical-chemical tests?


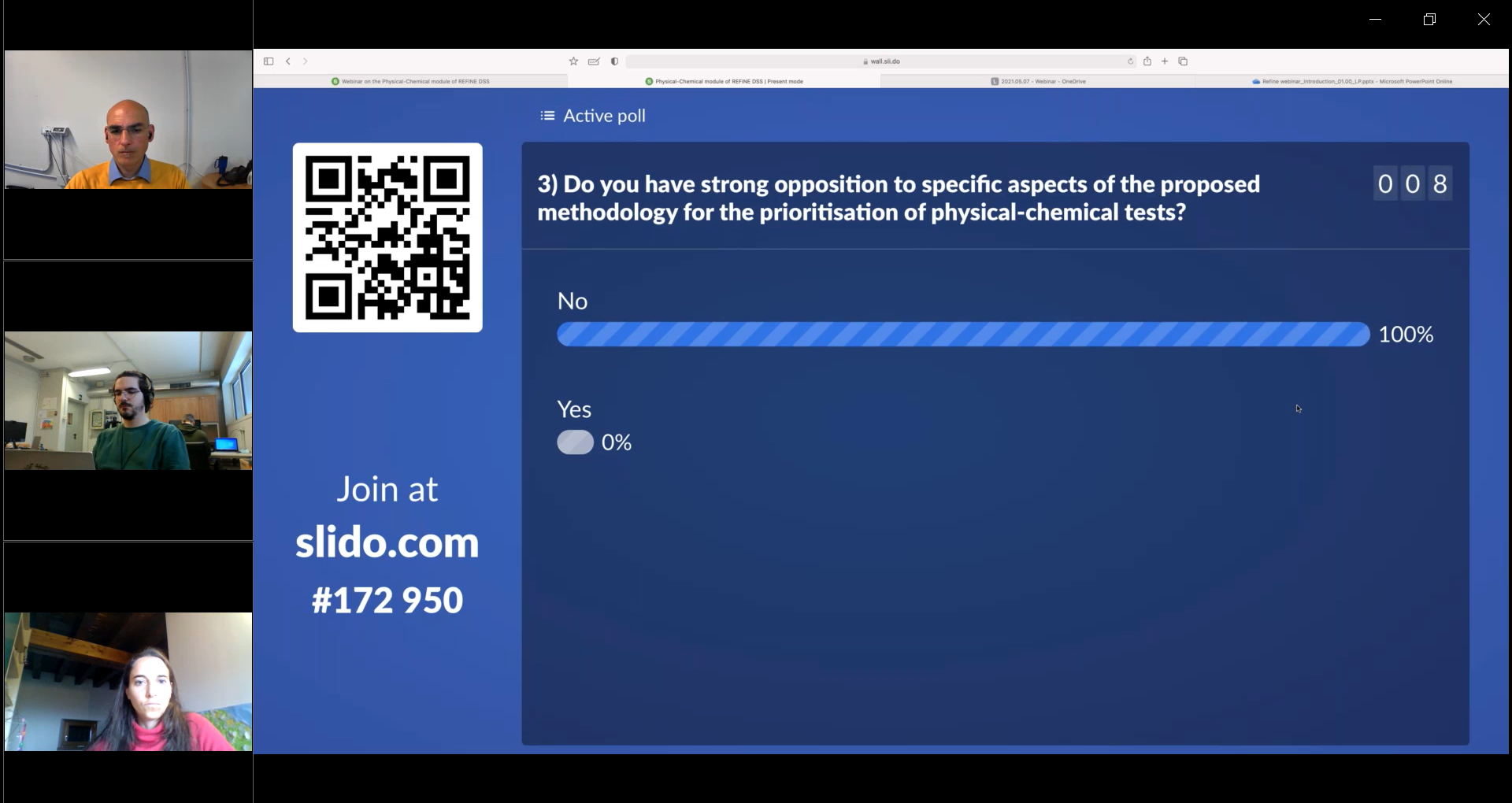


Figure 4. Answers to the third question

1. Do you think the access pages and the structure of the REFINE DSS are clear?


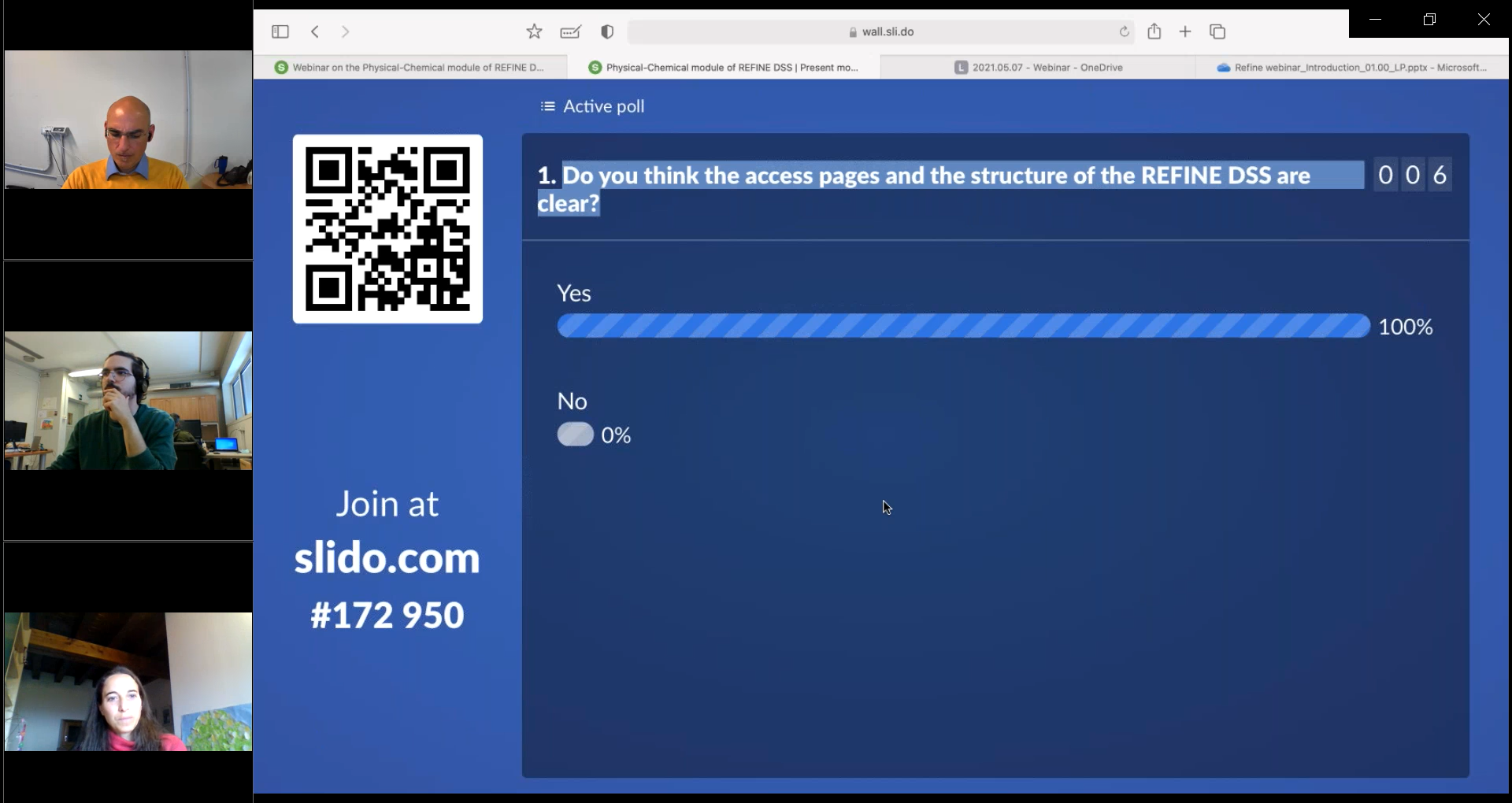


Figure 5. Answers to the fourth question

1. Who do you think the DSS should address as targeted users?


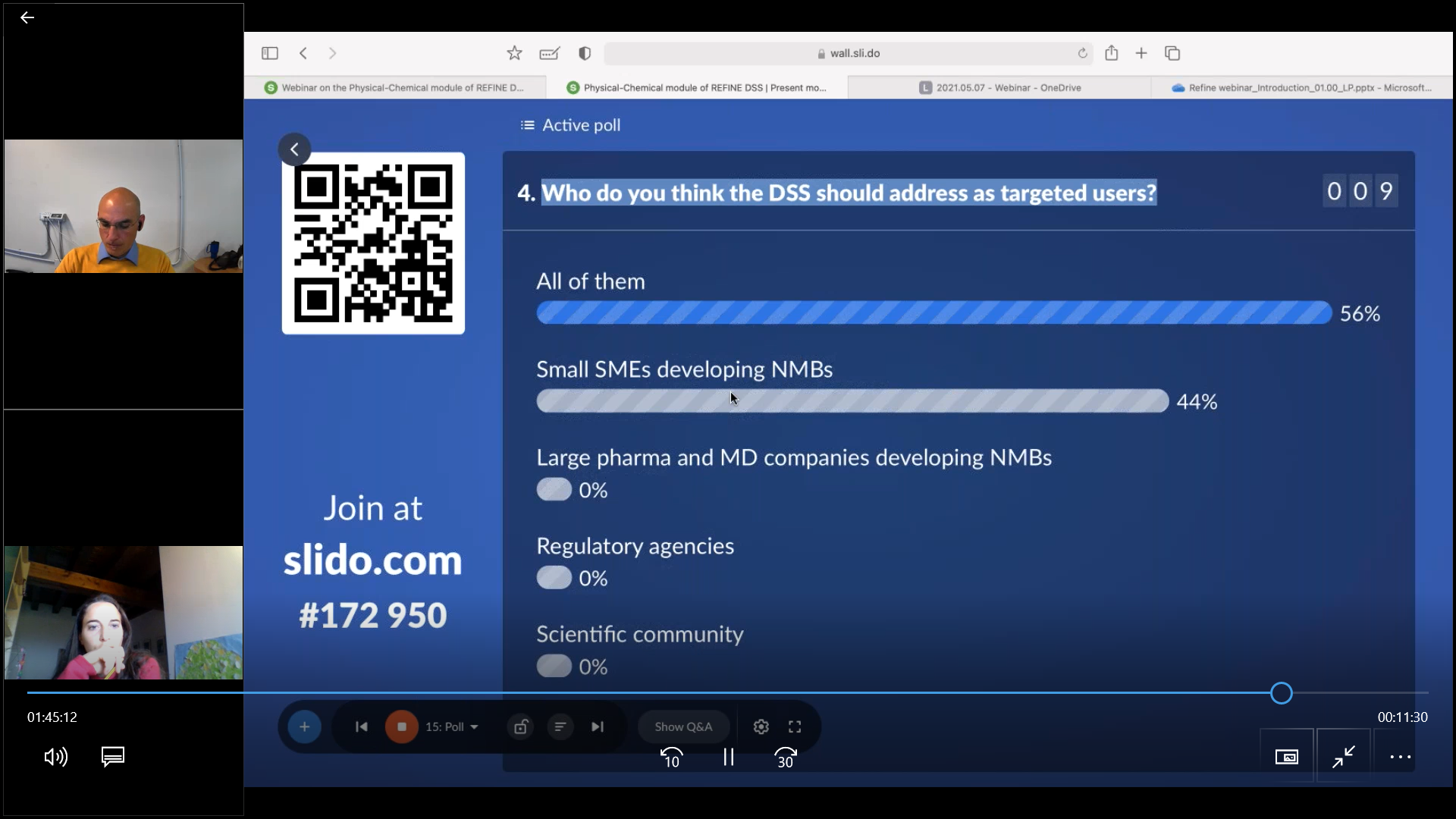

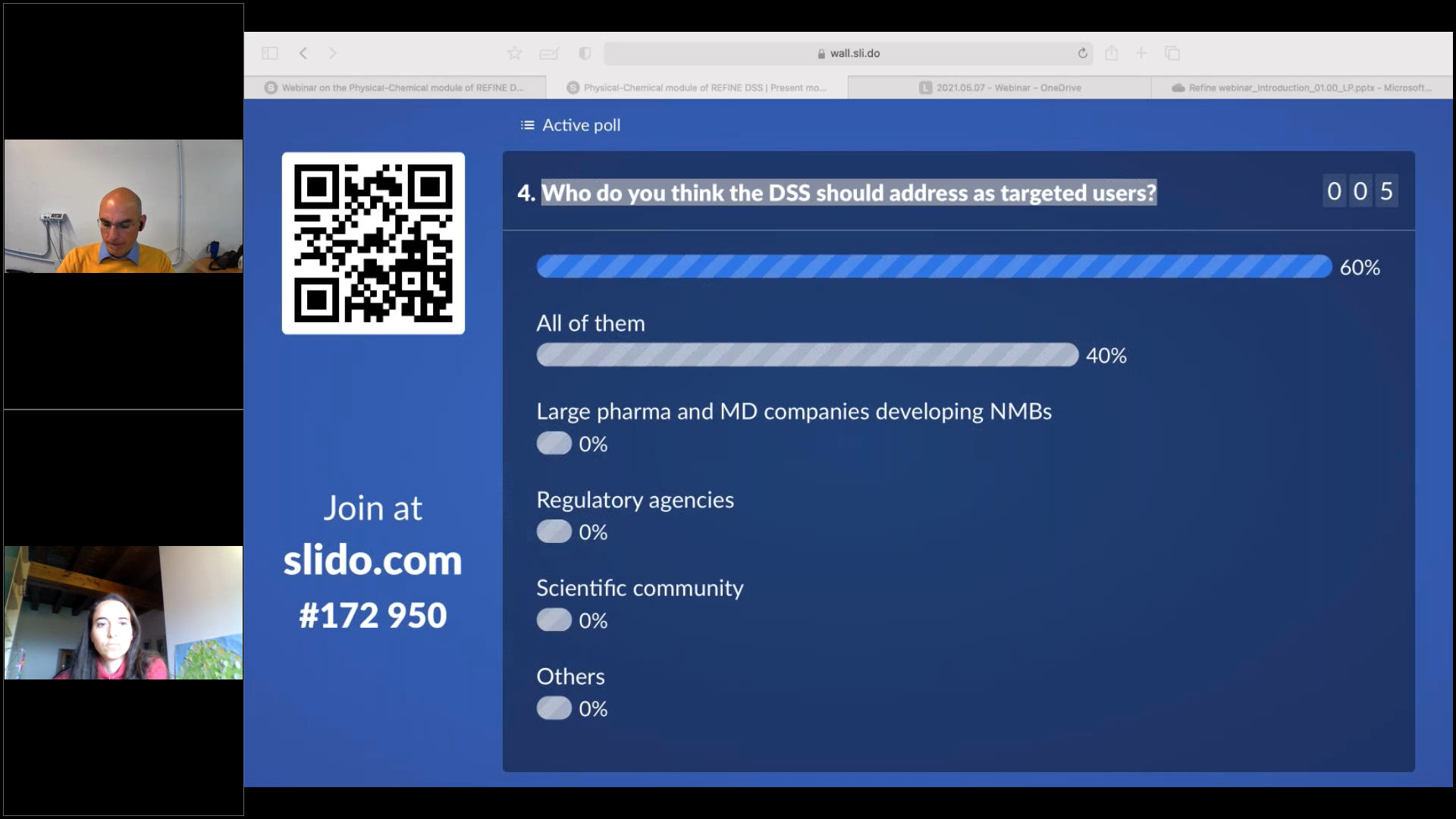


Figure 6. Answers to the fift question

1. What do you think are the main obstacles for the use of REFINE DSS?


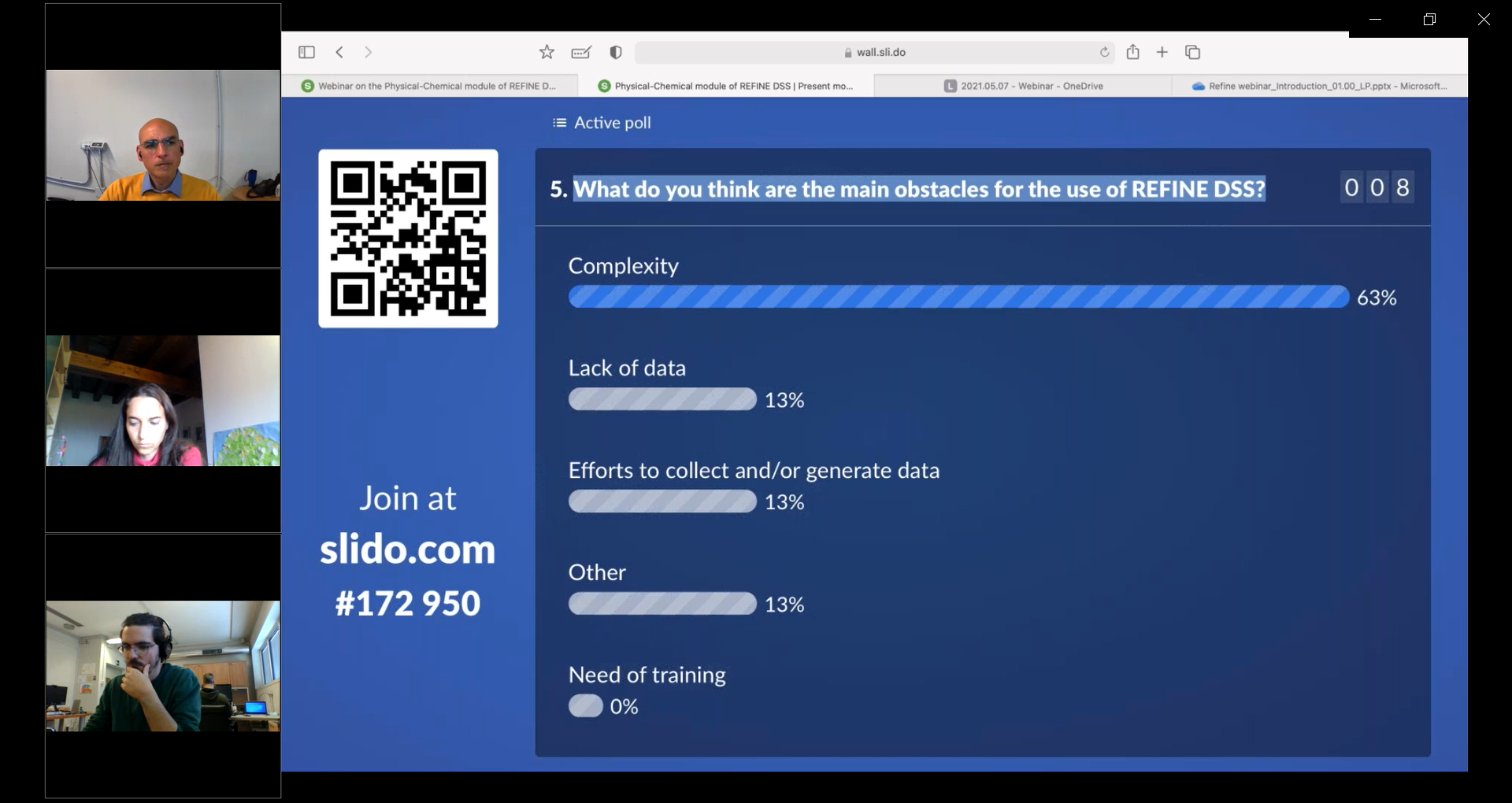


Figure 7. Answers to the sixt question.

# part 2: questions and answers for the Second External Webinar

1. Do you think that the developed modules of the REFINE DSS for the prioritisation of physical-chemical tests are suitable for the purpose?


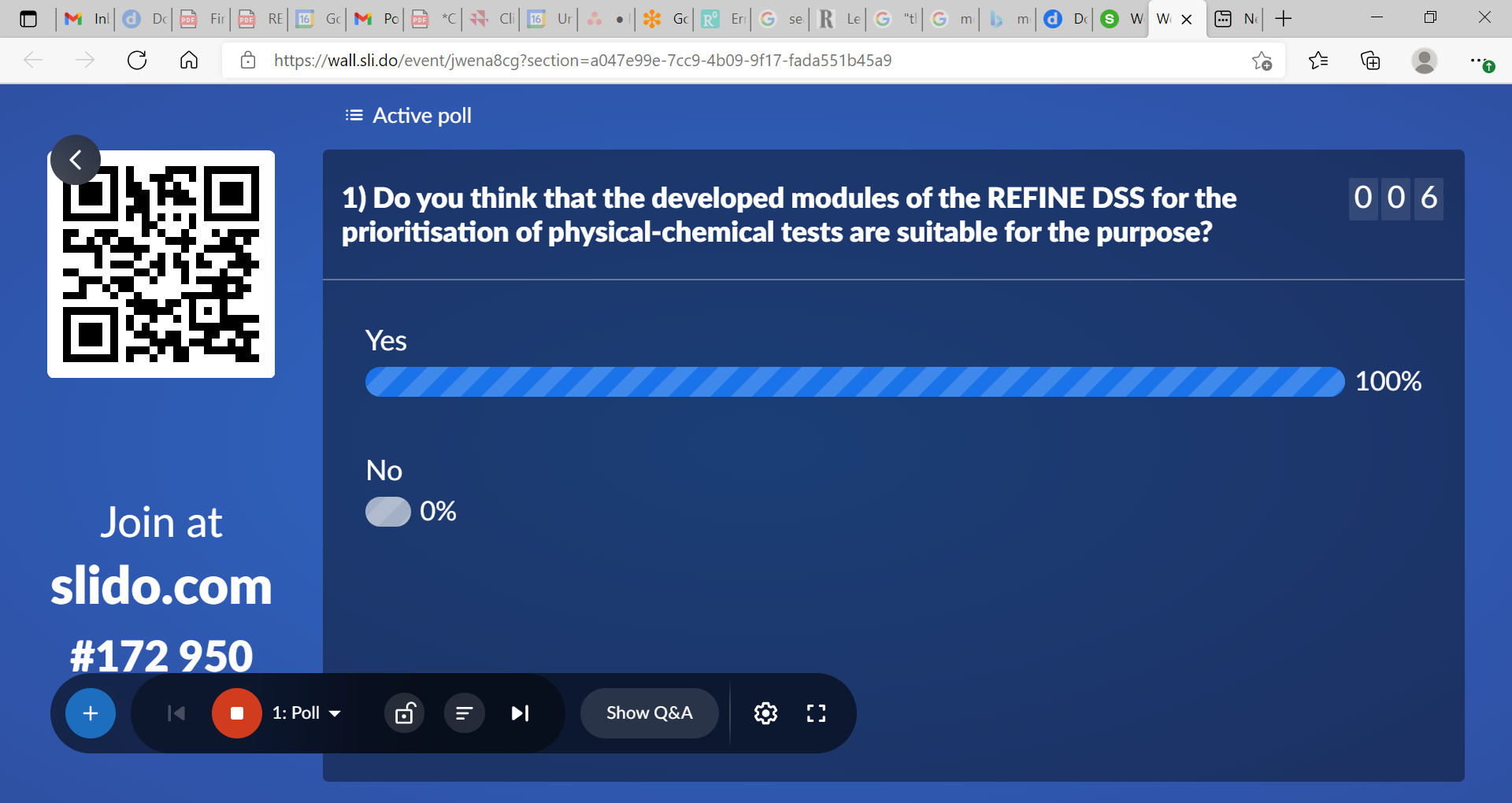


Figure 8. Answers to the seventh question

1. Can you identify additional aspects that need to be considered in the prioritisation?


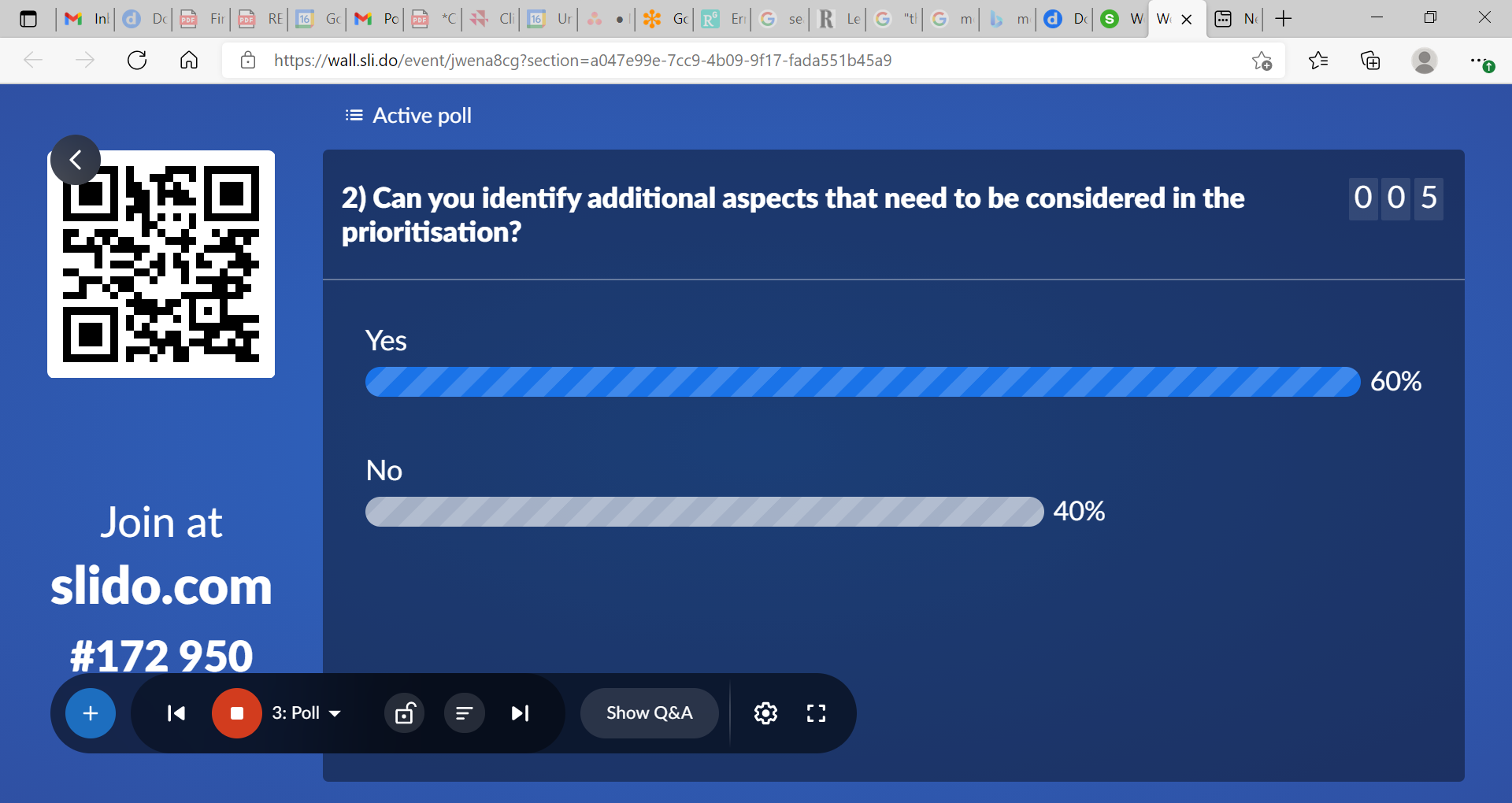


Figure 9. Answers to the eight question


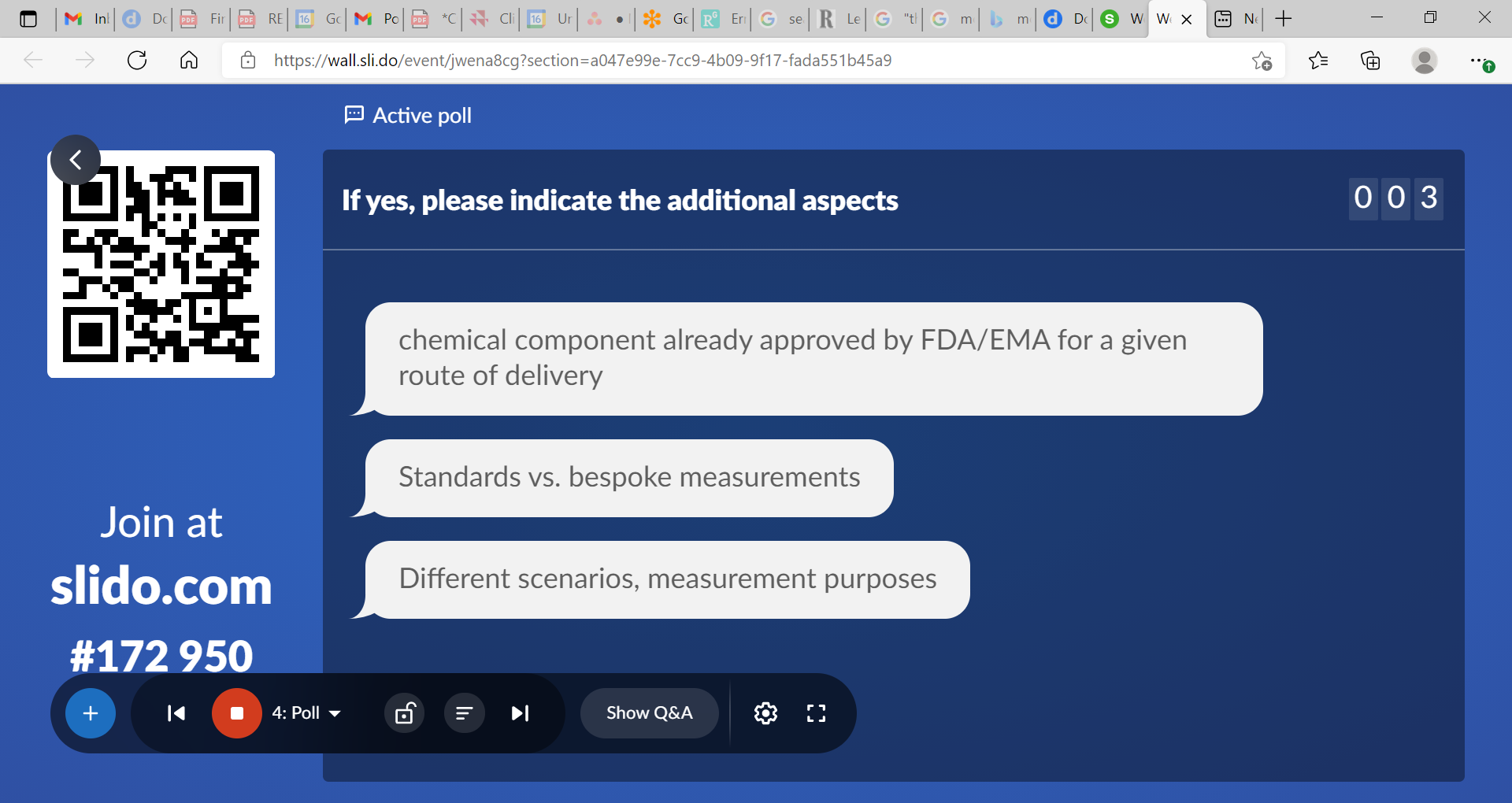


Figure 10. Additional aspects to be included in the REFINE DSS, as further explanation of the question in Figure 9

1. Do you have strong opposition to specific aspects presented today for the prioritisation of physical-chemical tests?


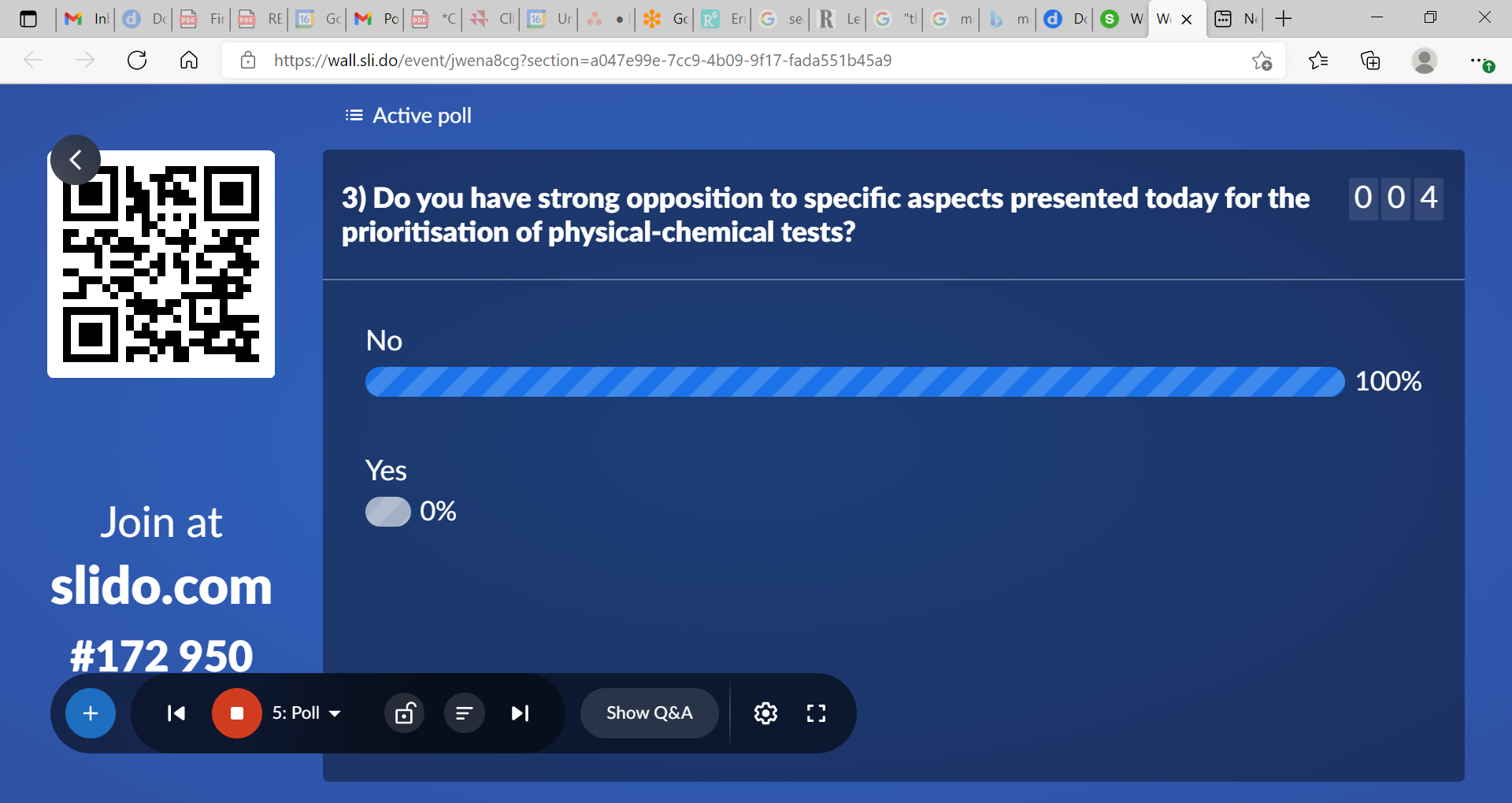


Figure 11. Answers to the night question

1. Was easy for you to understand how the DSS works?


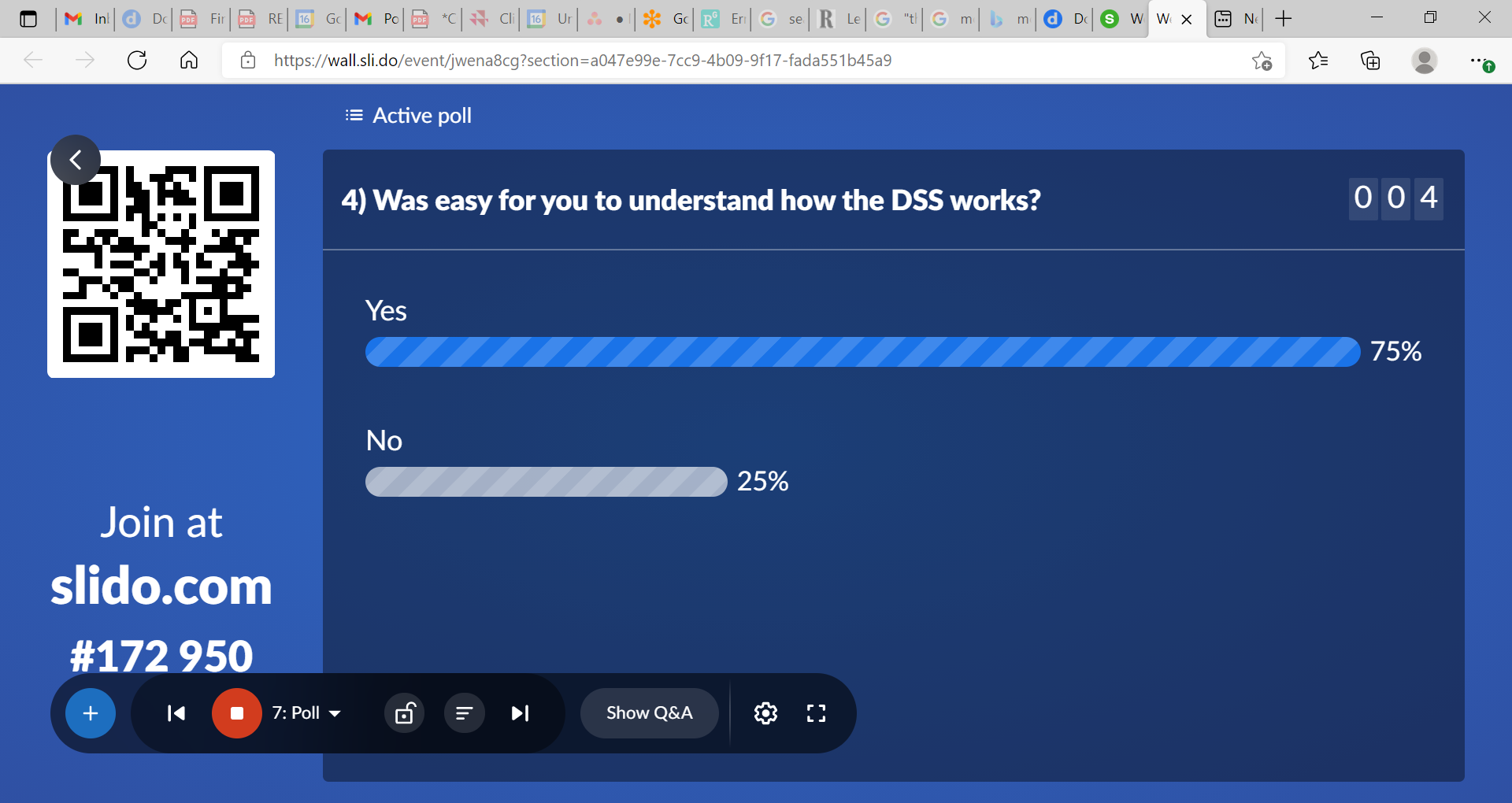


Figure 12. Answers to the tenth question

1. Do you think the access pages and the structure of the REFINE DSS are clear?


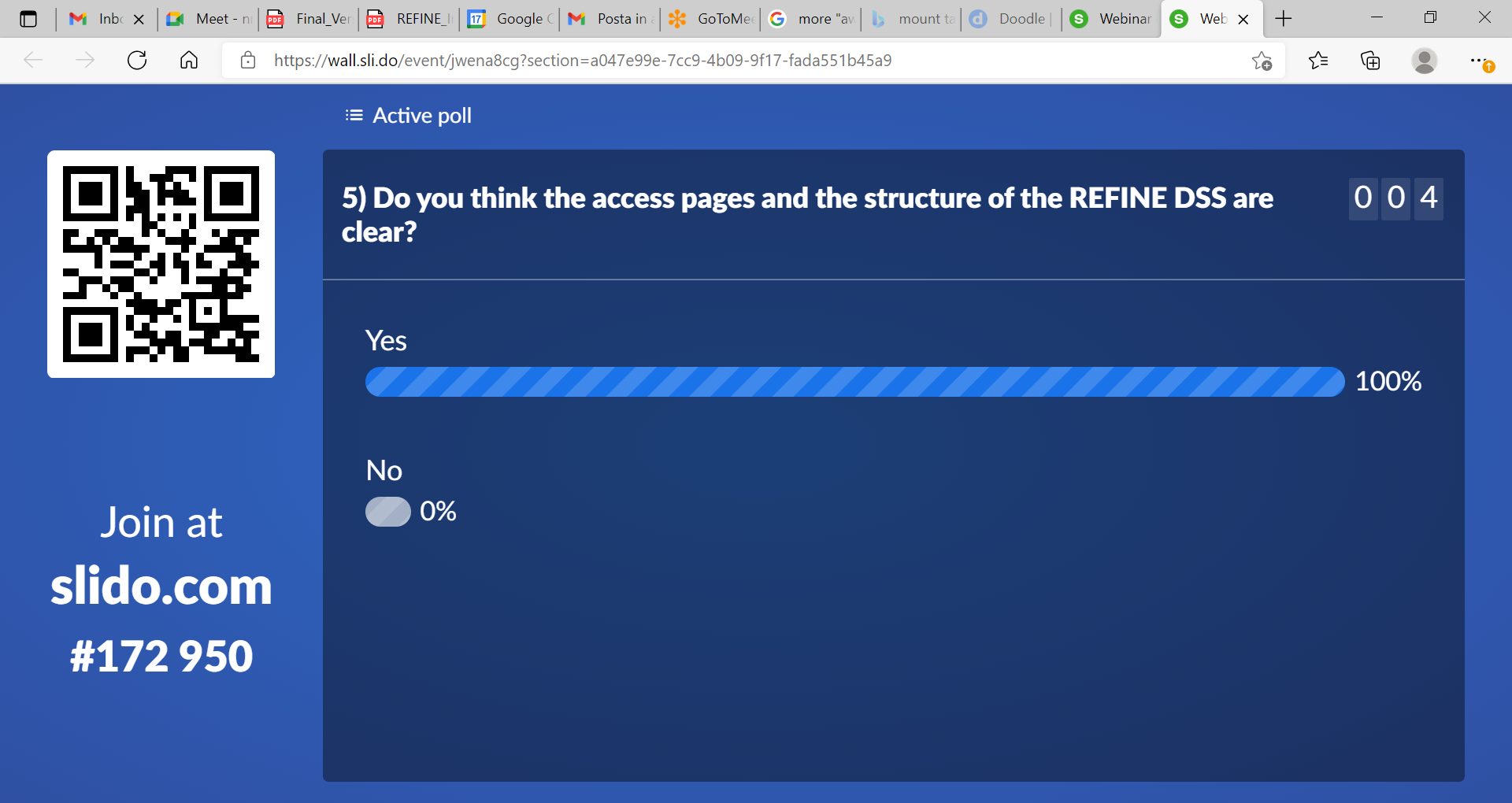


Figure 13. Answers to the eleventh question

1. Do you think that the results of the intelligent testing strategy are presented clearly and are easy to understand?


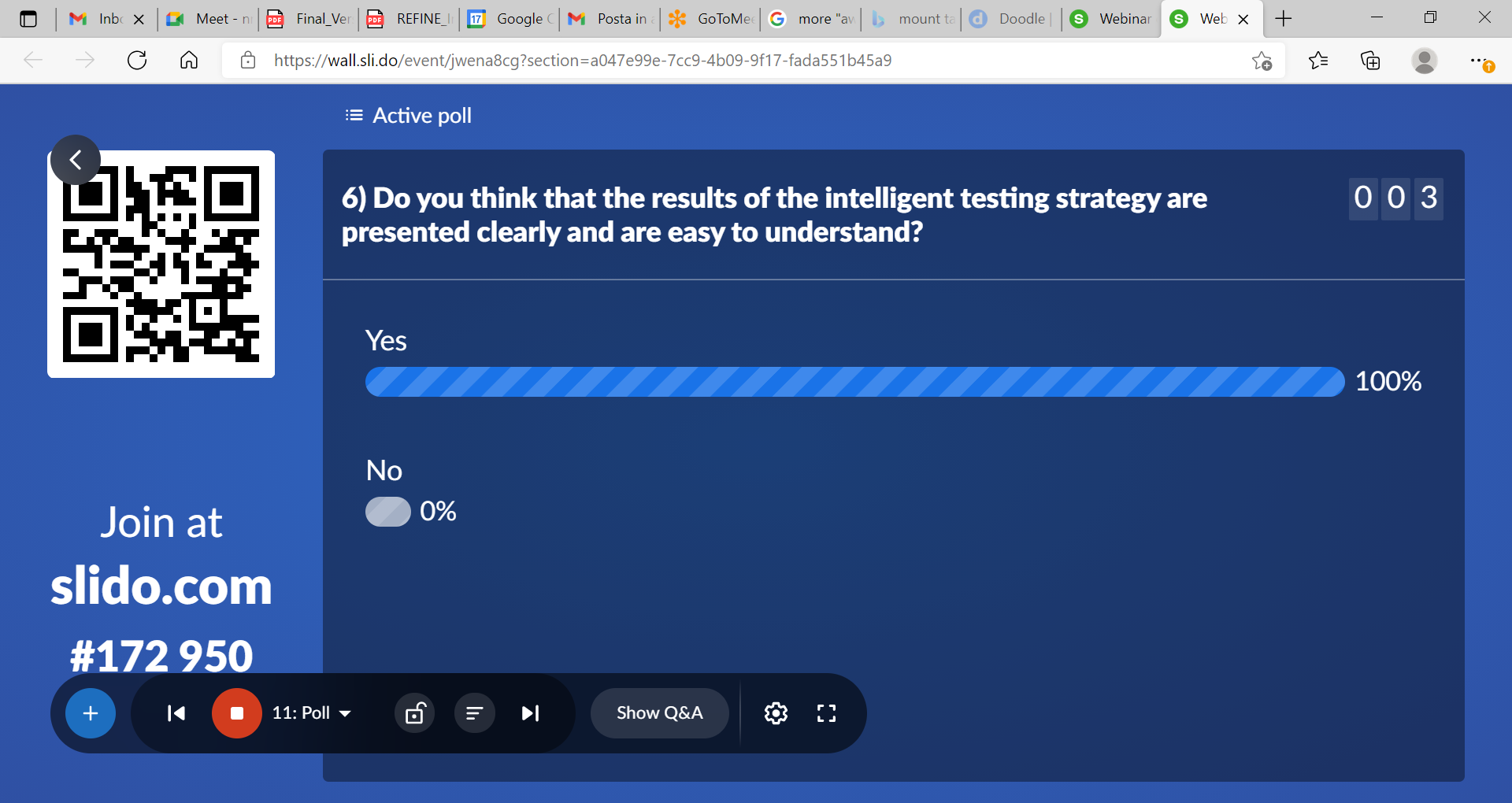


Figure 14. Answers to the twelfth question of the second webinar

1. Which aspect or functionality is missing in the current version of the REFINE DSS?


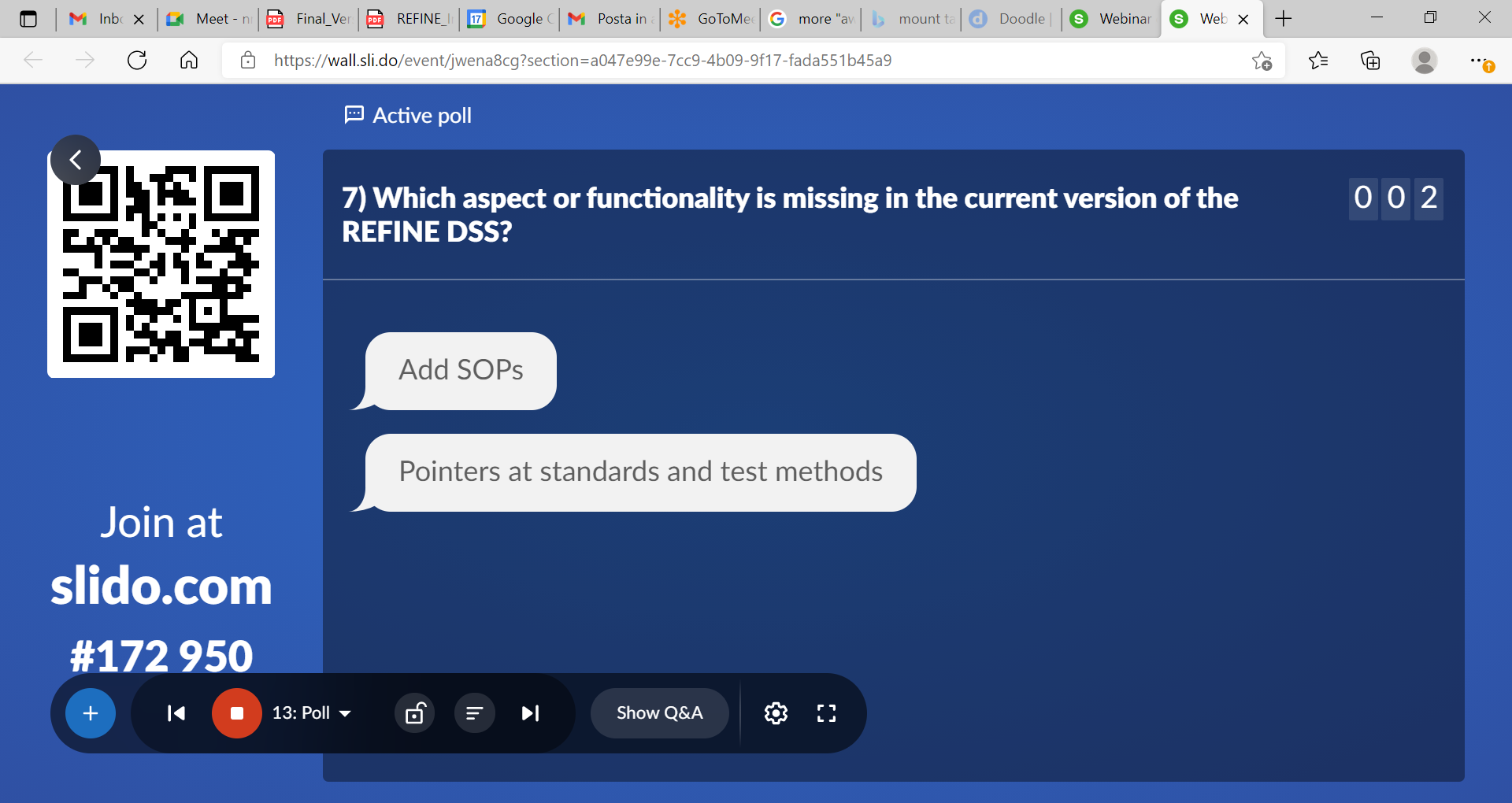


Figure 15. Answers to the thirteenth question

1. Who do you think the DSS should address as targeted users?


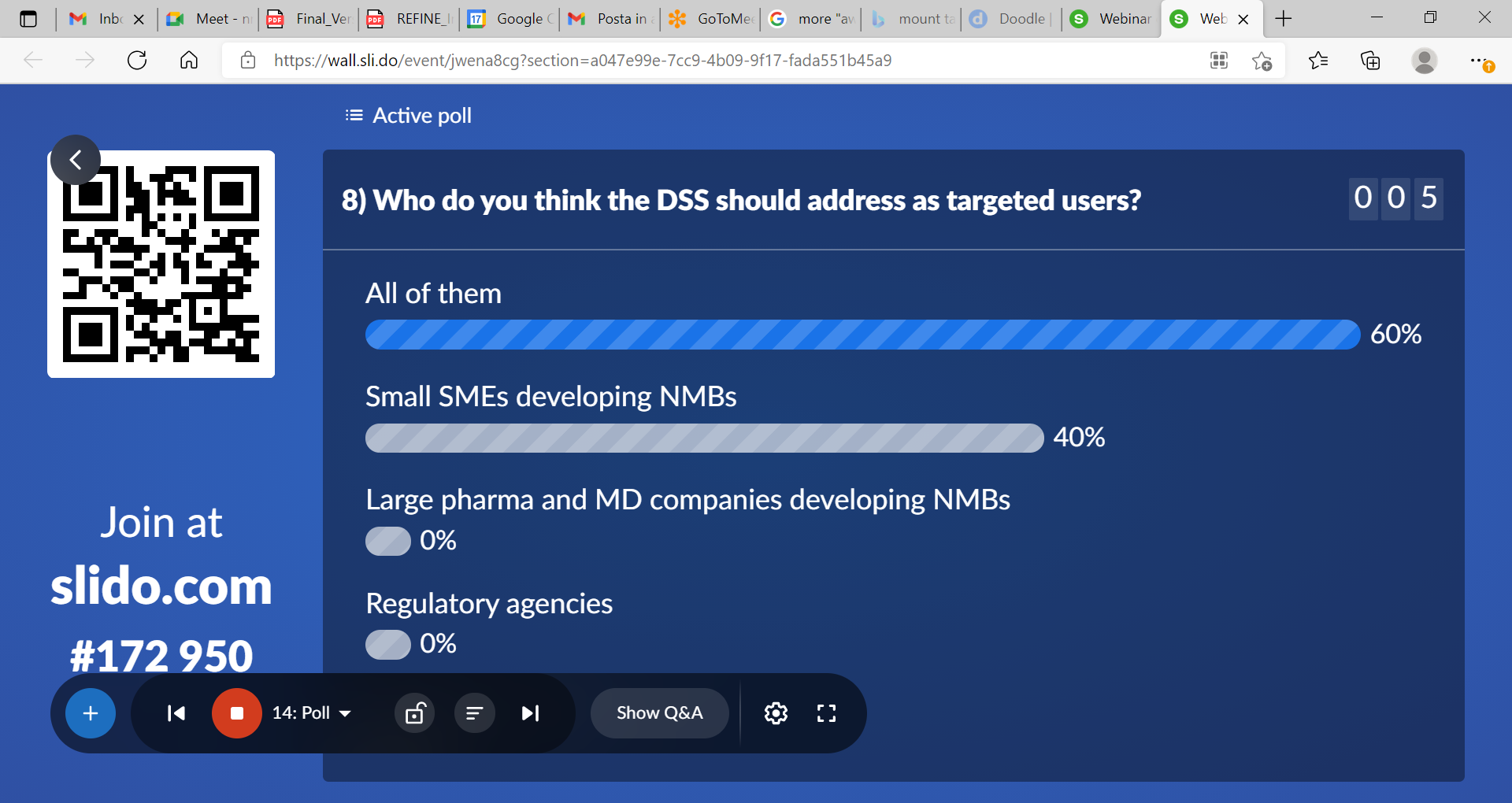

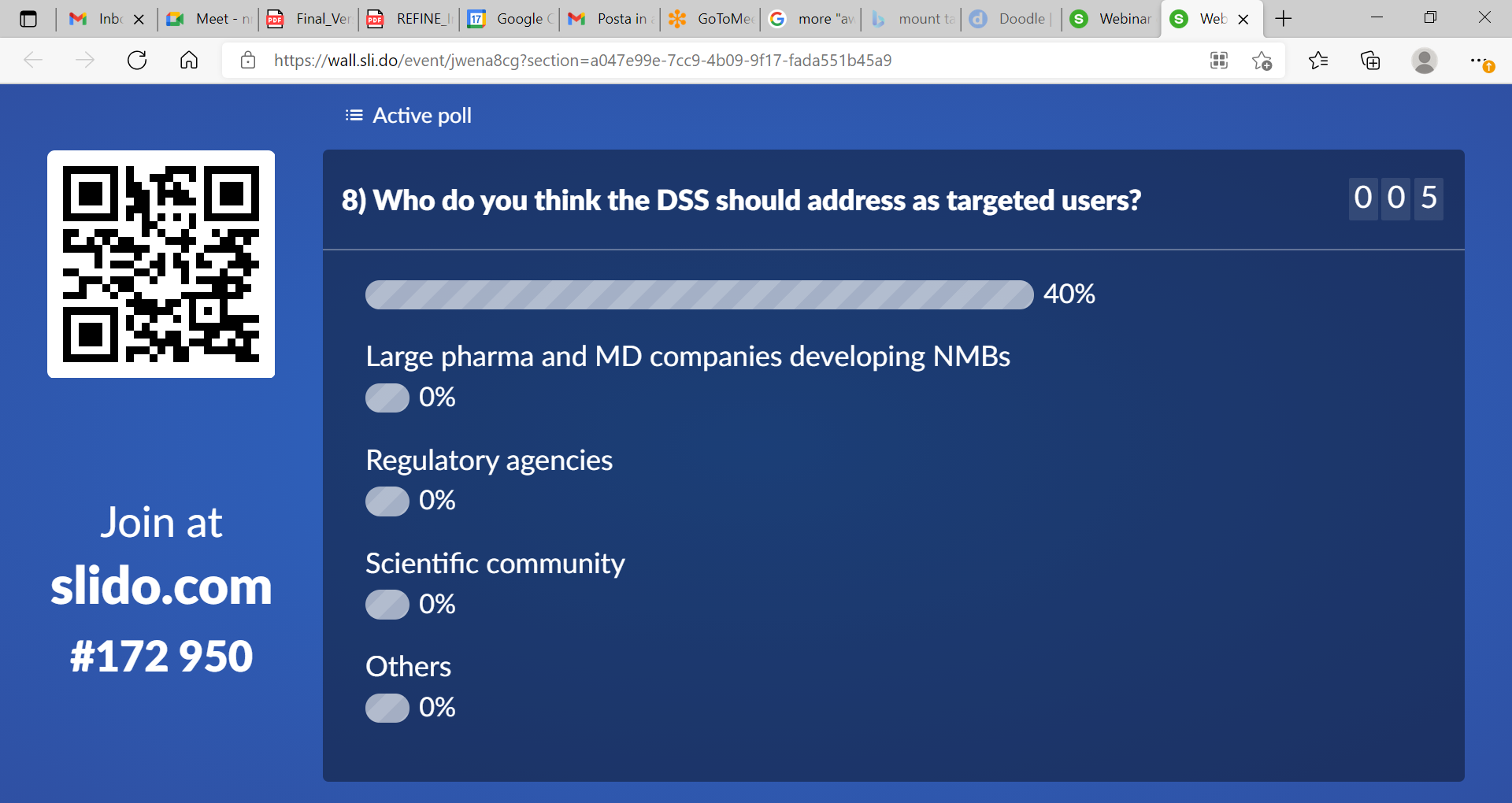


Figure 16. Answers to the fourteenth question

1. What do you think are the main obstacles for the use of REFINE DSS?


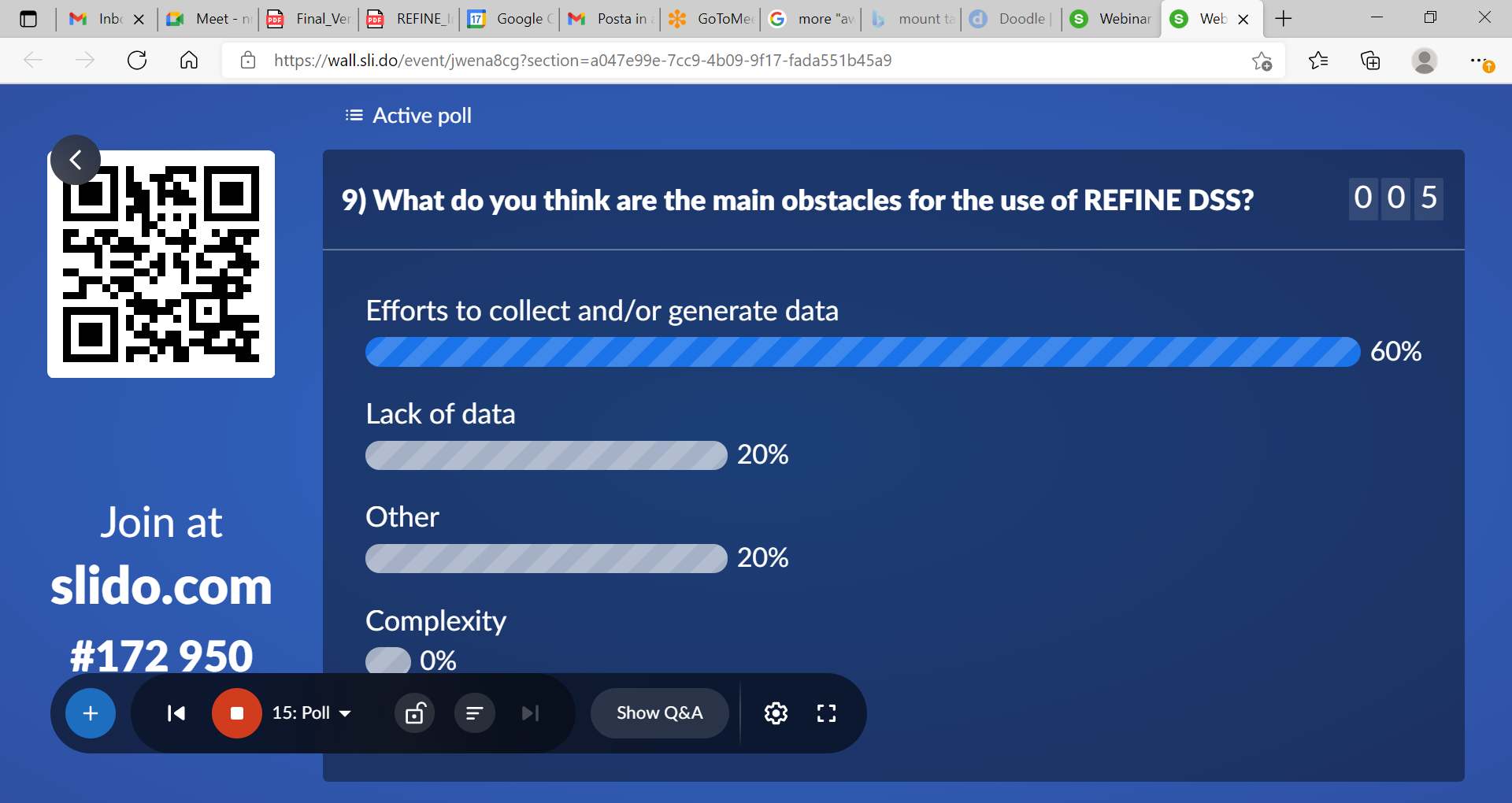

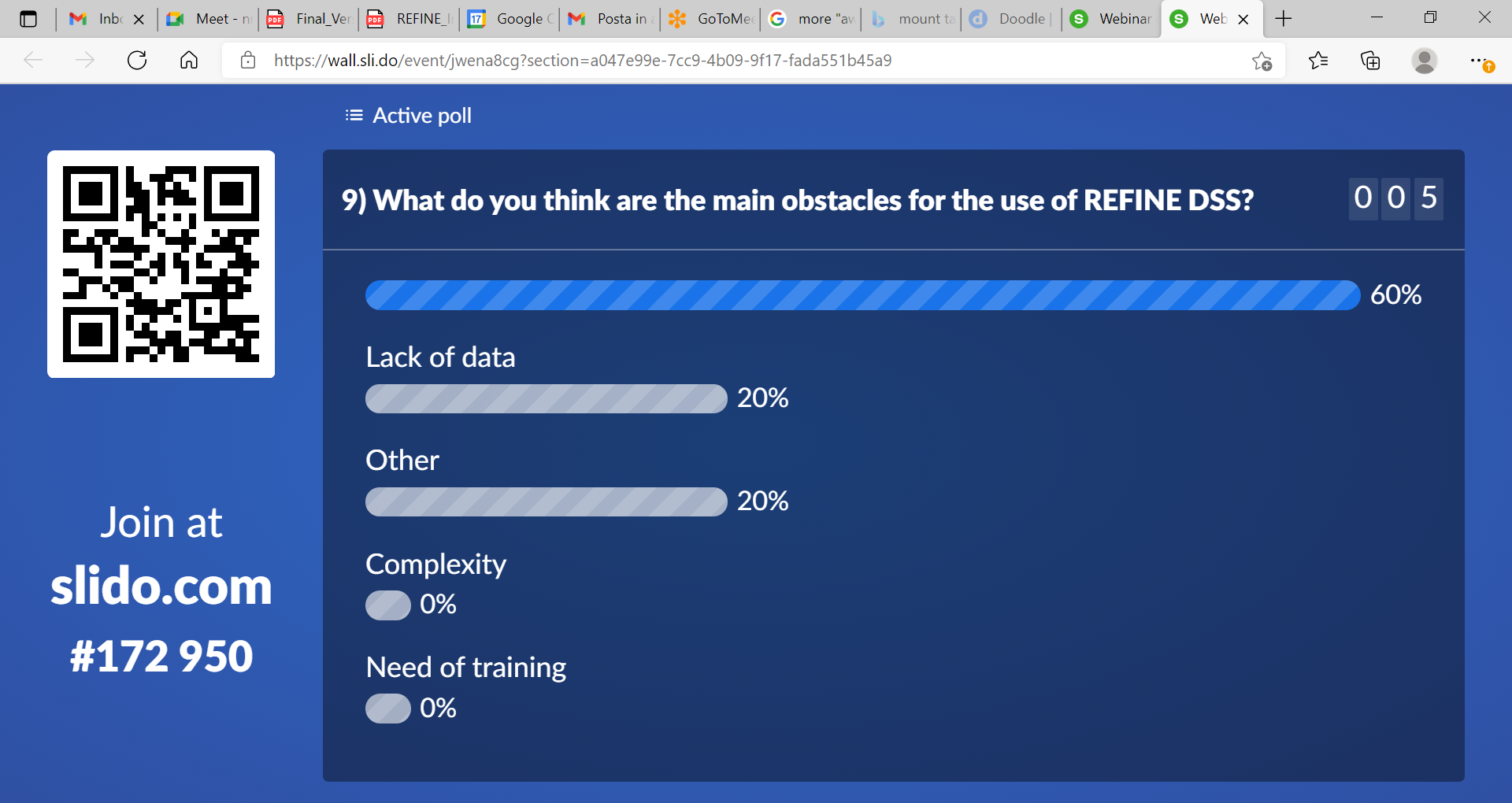


Figure 17. Answers to the fifteenth question
